# Long-Lived Photo-Response of Multi-Layer N-Doped Graphene-Based Films

**DOI:** 10.1021/acs.jpcc.3c04670

**Published:** 2023-08-30

**Authors:** Jokotadeola A. Odutola, Horatiu Szalad, Josep Albero, Hermenegildo García, Nikolai V. Tkachenko

**Affiliations:** † Photonics Compound and Nanomaterials (Chemistry and Advanced Materials Group), Faculty of Engineering and Natural Sciences, Tampere University, Korkeakoulunkatu 8, FI-33720 Tampere, Finland; ‡ Instituto Universitario de Tecnología Química, Universitat Politècnica de València, Avda. de los Naranjos s/n, 46022 Valencia, Spain

## Abstract

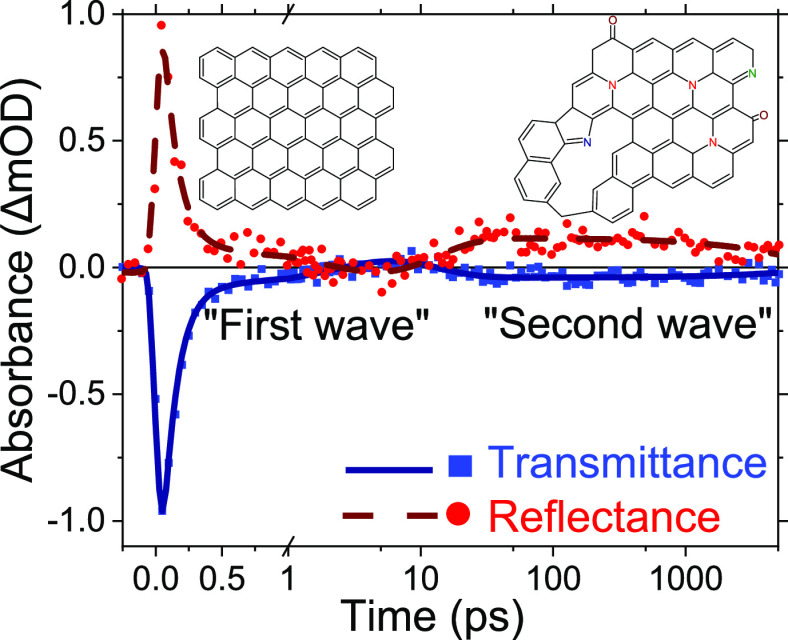

New insights into the mechanism of the improved photo(electro)catalytic activity of graphene by heteroatom doping were explored by transient transmittance and reflectance spectroscopy of multi-layer N-doped graphene-based samples on a quartz substrate prepared by chitosan pyrolysis in the temperature range 900–1200 °C compared to an undoped graphene control. All samples had an expected photo-response: fast relaxation (within 1 ps) due to decreased plasmon damping and increased conductivity. However, the N-doped graphenes had an additional transient absorption signal of roughly 10 times lower intensity, with 10–50 ps formation time and the lifetime extending into the nanosecond domain. These photo-induced responses were recalculated as (complex) dielectric function changes and decomposed into Drude–Lorentz parameters to derive the origin of the opto(electronic) responses. Consequently, the long-lived responses were revealed to have different dielectric function spectra from those of the short-lived responses, which was ultimately attributed to electron trapping at doping centers. These trapped electrons are presumed to be responsible for the improved catalytic activity of multi-layer N-doped graphene-based films compared to that of multi-layer undoped graphene-based films.

## Introduction

Graphene, a one-atom thick 2D material constituted by sp^2^-hybridized carbon atoms in a hexagonal arrangement, has recently become popular^1^ since it was first isolated by using tape to exfoliate highly oriented pyrolytic graphite.^2^ This is because of its unique properties, including high electric and thermal conductivity, mechanical and chemical resistance, and high specific surface area,.^3^ Owing to the newness of the field, there is a lack of consensus regarding the classification of graphenes, especially when differentiating them from graphite. The most common classifications are based on the number of graphene layers including terms such as monolayer, bilayer, and few-layer graphene.^3,4^ However, with several layers, a lack of agreement ensues with terms including multi-layer or thick graphene,^5^ ultrafine graphite,^6^ and graphite nanosheets,^7^ which renders any proper classification of the samples difficult. Therefore, a more literal or descriptive term “multi-layer graphene-based films” was utilized for this paper, with the definition of a multiple-layered film based on graphene sheets.

In electronics, high-quality, defect-free graphenes are utilized due to their exceptional electrical conductivity, which can surpass even metals. Such exceptional conductivity is attributed to its unique electronic structure, which as a function of energy and momentum can be represented by a Dirac cone.^8^ A Dirac cone consists of an unfilled π* band comprising the carbon anti-bonding orbitals and a filled π band comprising the carbon bonding orbitals, which meet at a Dirac point.^9^ At the Dirac point, carriers can move from the π to π* band, consequently providing a quasimetallic property to graphene. When increasing the number of layers from bilayer graphene to graphite, the π and π* bands become more parabolic.^10^

Other graphene applications, particularly catalysis which is the focus of our study, require lattice defects which promote semiconductor behavior.^11,12^ Such defects include carbon vacancies, five or seven carbon member rings or the substitution of carbon atoms by hetero-atoms, such as O, N, P, and B. Depending on the hetero-atom electro-negativity relative to C, the doping can be categorized as either n-doping with atoms such as N, O, and P or p-doping with atoms such as B. These hetero-atom-doped graphene films have been shown through density functional theory (DFT) simulations to have (opto) electronic properties distinct from those of undoped graphene films. Both n-doping and p-doping cause a separation between the bands at the Dirac point due to contributions from the hetero-atom p_*z*_ orbitals in addition to the carbon 2p_*z*_ orbitals from graphene.^8,13,14^ Also, the Dirac point relative to the Fermi level changes; n-doping causes a higher Fermi level compared to the Dirac point and p-doping causes a lower Fermi level compared to the Dirac point.^8,9,13,14^ However, a special case occurs for certain defects such as O-doping or vacancies leading to distorted five or nine carbon member rings, wherein the Dirac point opening is accompanied by the appearance of an extra band between the π and π* band.^15^ DFT calculations also revealed changes to the dielectric function of doped graphene compared to that of undoped graphene for the in-plane polarization of light such as dampening^8,9,14^ of the imaginary part at the peak at approx. 4.3 eV (corresponding to the π → π* transition) and shifts in the peak of the real part of the dielectric function with increased hetero-atom doping.^8^ In addition, vacancies in the graphene sheet were shown to cause the dampening of the peak of the imaginary part of the dielectric function at 4.3 eV.^15^

Defective graphenes demonstrate photocatalytic activity for hydrogen evolution,^16^ heterogeneous catalysis,^17^ electrocatalysis,^18^ and energy storage,^19^ among other applications.^20−23^ Despite this, compared to pristine graphene^24,25^ or even graphite,^26,27^ few photophysical studies particularly using optical pump–optical probe spectroscopy have been conducted on doped graphenes. Kadi et al. performed a microscopic study of the carrier dynamics in n- and p-doped graphene.^28^ Compared to that in p-doped graphene, the carrier thermalization in n-doped graphene was faster due to more available scattering partners in the conduction band. Similar results were also reported by Johannsen et al.^29^ These reports show how doping effectively shifts the photoresponse of graphene in subpicosecond to picosecond time domains; however, such short time scales are unlikely to affect the catalytic activity. A long-lived transient at around 300 nm was observed by Oum et al.^30^ but was attributed to the interfacial heat flow from the graphene to the supporting substrate. Considering the difference in opto (electronic) properties and the changes in the transient response at shorter time scales (subpicosecond to picosecond) of doped graphene compared to those of undoped graphene, the transient response at longer time scales (>1 ns) requires further investigation. Presumably, the photoexcitation of doped graphene should activate the photo-catalytic centers with sufficient lifetime for interfacial chemical reactions, which have not yet been observed using time-resolved spectroscopy.

Consequently, the opto (electronic) response of multi-layer N-doped graphene-based films pyrolyzed from chitosan^31^ on a quartz substrate was studied in this paper using transient transmittance and reflectance spectroscopy and compared to those of a multi-layer undoped graphene-based film control. As with several other spectroscopic studies,^25,30,32^ multi-layer graphene-based films were used in this paper instead of monolayer graphene, which is barely sensitive with the optical pump–probe technique because of the monolayer’s high transparency in the visible range with transmittance essentially over 90%.^30^ However, attention was paid to using advanced characterization techniques to ensure that the samples in the study were indeed multi-layer graphene-based films and not “pure” graphite films. The multi-layer N-doped graphene-based films were prepared as a series with different nitrogen and surface defect proportions as a function of pyrolysis temperature, 900–1200 °C,^33^ with a targeted optical density of 0.2–0.4 at the excitation wavelength (500 nm). Then, the resulting optical spectra were interpreted by data modeling using Drude–Lorentz (D–L) parameters^34,35^ to decode the carrier dynamics responsible for the improved catalytic activity of multi-layer N-doped graphene-based films.

## Experimental Section

### Materials

Multi-layer defective, N-doped graphene-based films were prepared from spin-coated chitosan aqueous solutions on a quartz substrate and pyrolyzed under argon at four different temperatures (900, 1000, 1100, and 1200 °C; see Section S1). The chitosan concentrations were adjusted to obtain films with transmittance suitable for transient absorption (TA) studies and labeled as NGF900, NGF1000, NGF1100, and NGF1200. A multi-layer undoped graphene-based control film (labeled as GTF) was prepared by polystyrene sublimation on quartz substrate as previously reported.^36^

### Instrumentation

The Raman spectra were acquired using a Horiba Jobin Yvon-Labram HR UV–Visible– near-infrared Raman microscope spectrometer, using a laser at 632 nm excitation.

The X-ray photoelectron spectroscopy (XPS) spectra were acquired using a SPECS spectrometer (Surface Nano Analysis GmbH, Berlin, Germany) with a Phoibos 150 MCD-9 detector. Before measurements, the samples were evacuated into the XPS setup antechamber at 10^–9^ mbar (see Section S1 for further details).

The atomic force microscopy (AFM) images were acquired using a Veeco AFM apparatus with the contact mode at ambient temperature in air to measure the thickness and roughness. The films were scratched to determine the thicknesses (see Section S1 for further details).

The steady-state transmittance, T, of the samples was measured with a Shimadzu UV-3600 series spectrophotometer in air. The specular reflectance, R, of the samples was measured with the same spectrometer and a specular reflectance attachment (for 5° incidence angle) in air (see Section S1 for further details).

The transient absorption spectra were acquired using a laser pump–probe setup. The fundamental laser pulses at a repetition rate of 1 kHz and a pulse width of 100 fs were generated at 800 nm by the Libra F system, Coherent Inc., which was coupled with an optical parametric amplifier (OPA) Topas C, Light Conversion Ltd. These laser pulses were used to produce the pump beam to excite the sample and the probe beam (white continuum) to monitor the spectra. The pump beam wavelength at 500 nm (0.1 mJ cm^–2^) was generated by channeling a portion of the fundamental laser to the OPA. The white light for the monitoring range of 530–1100 nm was generated by channeling the rest of the light to a sapphire crystal. The transient absorption responses of the probe beam which were facilitated by means of a delay line were then measured using an ExciPro TA spectrometer (CDP, Inc.) with the Si and InGaAs diode arrays for the visible and the NIR ranges, respectively. An in-house-developed fit program was used for the multiexponential fitting of the transient responses for all the samples with the instrument response (100 fs) modeled by a Gaussian pulse and group velocity dispersion compensation (see Section S1 for further details).

## Results and Discussion

### Sample Structure Characterization

The formation of multi-layer defective graphene-based films with graphitic (G) and defect (D) bands at 1590 and 1350 cm^–1^, respectively, was confirmed by Raman spectroscopy (see Figure S2). The *I*_G_/*I*_D_ ratios in the NGF900–1200 films, with values of 0.84, 0.85, 0.80, and 0.81, illustrate a roughly similar defect proportion.

The C 1s, O 1s, and N 1s high-resolution composition spectra obtained by XPS are presented in Figure S3 (see Section S2 for further discussion). The C, N, and O proportions and chemical environments are summarized in Tables 1 and S1, respectively. For the comparisons, we assumed the homogeneity of the multi-layer N-doped graphene-based films in the sense of their breadths, as XPS is only a surface characterization technique. As previously reported,^33^ the temperature promotes a decrease in N content (pyridinic-N reduces, while the N-oxides increases) and increase in graphitization (sp^2^ component of the C 1s peaks).^33^ Conversely, the trend for the O content from the incomplete carbonization of the chitosan precursor fluctuated within a particular range, although there was an overall decrease from NGF900 to NGF1100. However, we can infer from these results in combination with the Raman spectra that increased pyrolysis temperatures reduces the number of defects or promote graphitization in multi-layer defective, N-doped graphene-based films. Nevertheless, the XPS measurements (shown in Figure S3) do not indicate the detectable presence of any sp^3^ bands corresponding to graphite,^37^ meaning that the samples can be classified as multi-layer graphene films rather than graphite films. The trend in Table 1 is smooth at NGF900–1100 but “broken” at NGF1200 due to organic matter volatilization occurring over further chemical transformation.

**Table 1:** Summary of the Atomic Composition of the Samples Obtained by XPS

	loading	NGF900 (%)	NGF1000 (%)	NGF1100 (%)	NGF1200 (%)
C 1s	% wt	89.87	90.19	92.89	92.67
	% atom	91.86	92.22	94.36	94.25
O 1s	% wt	6.79	7.47	5.03	5.95
	% atom	5.21	5.74	3.84	4.54
N 1s	% wt	3.34	2.33	2.07	1.38
	% atom	2.93	2.03	1.81	1.20

The roughness and thickness of the multi-layer graphene-based films determined by AFM are shown in Figure S4 (see Section S2 for further discussion). Six independent cross-section measurements on the images of NGF900–1200 reveal average film thicknesses of 29.3 ± 5.7, 20.5 ± 1.7, 30.3 ± 2.4, and 45.3 ± 3.5 nm, respectively. The measured roughness mean square (*R*_q_) of all the samples is approximately 1.5 nm, indicating very flat and homogeneous surfaced films.

These characterization techniques confirm the production of multi-layer N-doped (defective) graphene-based films and as such a deviation in the opto-electronic properties of our NGF samples compared to the GTF reference and pristine monolayer graphene films in general is expected.

### Steady-State Spectra

**Figure 1:**
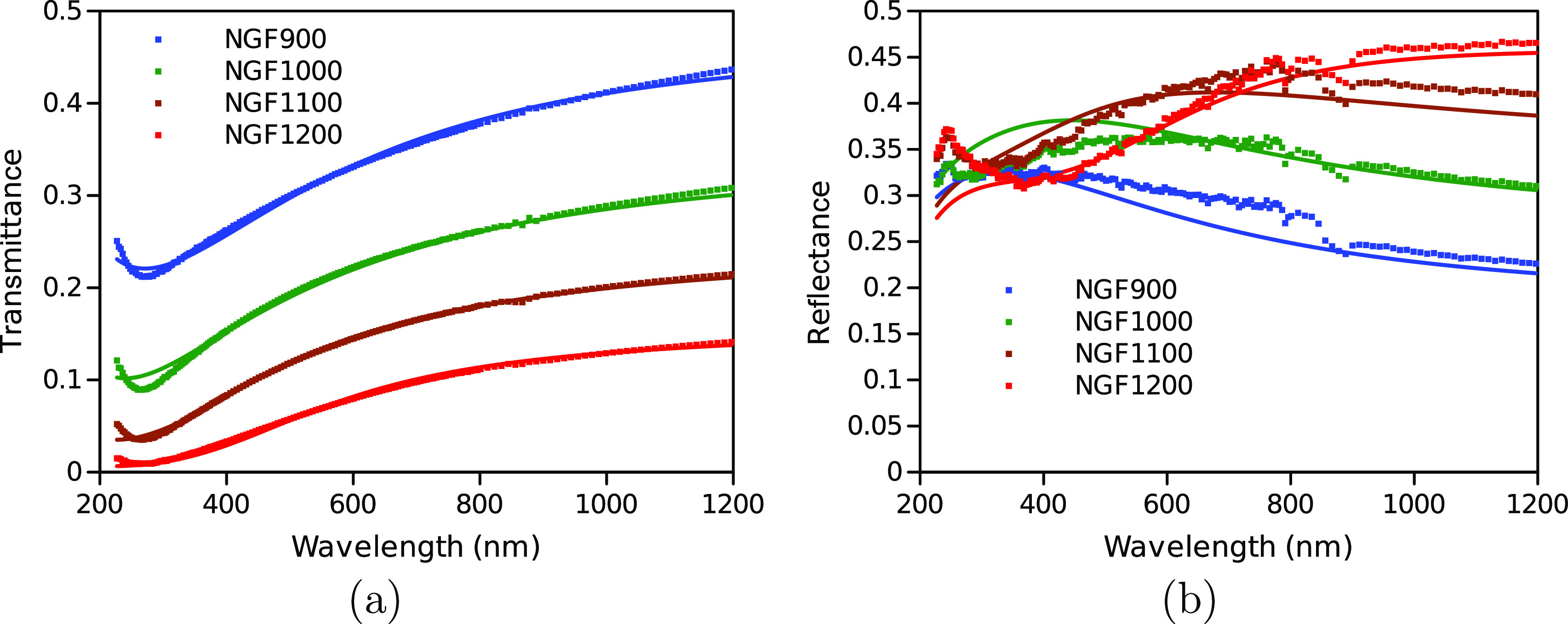
(a) Transmittance and (b) reflectance spectra of NGF900–1200 samples. The symbols are the measured raw data, and the lines are the global fitting results.

The sample steady-state transmittance (T) and reflectance (R) spectra are presented in Figure 1. The optical properties of the multi-layer graphene-based films in the UV–visible–near-infrared region were successfully modeled in frame of the Drude–Lorentz (D–L) dispersion model,^34,35^ including multi-layer-doped graphene-based films.^38^ The Drude component presents dielectric properties of the free electrons (plasmons), and the Lorentz band is due to π → π* transition. There are a few mathematically equivalent presentations of the D–L model.^39−43^ In this paper, we will use the following equation for the complex dielectric function based on the D–L dispersion model
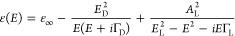
1where *E* = *h*ν is the photon energy, ε_∞_ is the high-frequency dielectric constant, *E*_D_ and Γ_D_ are the energy and damping of the Drude component, and *A*_L_, *E*_L_ and Γ_L_ are the intensity factor, the resonance energy, and the width (damping) of the Lorentz band, respectively. The amplitude of the Lorentz component, *A*_L_^2^, is sometimes presented as *A*_L_^2^ = *fE*_L_^2^, in which case *f* is referred to as the oscillator strength.^41,43^ In frame of the classic electrodynamics, the Drude frequency, ω_D_ = *E*_D_/ℏ, is determined by the density, *N*_e_, and effective mass, *m*_e_*, of the free carriers, electrons, as .^44^

It is notable that the transmittance spectra in Figure 1a show no specific absorption bands, which can be attributed to the doping centers. Therefore, the assumption is that the doping level is relatively low with no significant effect on the steady-state T and R spectra, which means that the dielectric function spectra are not affected significantly by the doping in the visible–NIR part of the spectrum. In other words, the differences in the T and R spectra between the samples come from the differences in the thicknesses and not from the dielectric function.

The experimentally measured spectra, T and R, are modeled using the transfer matrix method (TMM),^45,46^ which accounts for the light reflectance on both sides of the film as well as light interference within the film. These calculations are carried out using a known thickness *d* and the complex refractive index *ñ*. The latter is directly calculated from the dielectric function, . The spectra of all the doped samples were fitted globally to a common set of D–L parameters, which means that all the samples have the same dielectric function and individual film thicknesses. The measured and fitted spectra are shown in Figure 1, and fit details are provided in Section S3.1. There were two samples for each of the four pyrolysis temperatures. The difference between the samples pyrolyzed at the same temperature was minor; therefore, only one sample spectrum for each temperature is shown in Figure 1.

The samples were prepared specifically for TA measurements, which impose restrictions on the sample absorbance between 0.2 and 0.7 O.D within the monitoring range (530–1100 nm). Therefore, a different amount of precursor was used for samples annealed at different temperatures. The non-intended result was that the absorbance of the samples annealed at higher temperatures was higher. This trend is obvious from the T spectra in Figure 1a and AFM measurements in Figure S4.

The low accuracy of the measured reflectance spectra (Figure 1b) was due to the reference mirror corrections (see Section S1 for further discussion). Therefore, during the fit, the reflectance data was used with a weight factor of 0.1 relative to the transmittance data. However, the general trend in the reflectance spectra was consistent between the measured and modeled reflectance spectra at wavelengths >500 nm. Conversely, the region with the most apparent deviation of the fit curves from the data was the UV region, 230–350 nm. This is the region with the Lorentz band maximum, and apparently, a single Lorentz component is insufficient for the perfect spectra fitting. However, within the range of the TA measurements, roughly 530–1100 nm, the fits are reasonably good with deviations of less than 0.003 for the T spectra.

The D–L fit parameters are summarized in Table 2 and the sample thicknesses in Table S2. During the fit, the *E*_D_ value was fixed because of almost 100% correlation with Γ_D_, and *E*_L_ ended at the allowed lower limit of 4.4 eV (282 nm). The sample thicknesses obtained from transmittance and reflectance spectra measurements (Table S2) are of the same order of magnitude with the thicknesses estimated from the AFM measurements. However, there are some variations between these values due to the experimental error of measuring ultra-thin layers with AFM as explained in Section S3.2. Ultimately, the optically derived thicknesses were utilized for our analysis because first, they are taken from a larger and more representative multi-layer graphene-based film area (of a few squared millimeters), second, they relied on the same optical properties of the samples used in the analysis and, finally, they were of the same order of magnitude with the AFM values, which suggested a reasonably accurate fit.

**Table 2:** Drude–Lorentz Parameters Modeling the Steady-State Transmittance and Reflectance Spectra

parameter	value	comments
ε_∞_	2.1 ± 0.1	
*E*_D_, eV	6.0	fixed
Γ_D_, eV	3.4 ± 0.1	
*A*_L_, eV	12.2 ± 0.1	
*E*_L_, eV	4.4	at lower limit
Γ_L_, eV	6.7 ± 0.1	

The model spectra of real and imaginary parts of the dielectric function are shown in Figure S5. The obtained D–L model parameters were in good agreement with the previously reported values for multi-layer graphene films (see Figure S6 in Section S3.1).^34^

### TA Spectra

The TA measurements were carried out using a standard pump–probe technique, but complementing the detection of the standard transmitted probe (transient transmittance, or TT) with detection of the reflected probe (transient reflectance, or TR) as well, as schematically presented in Figure S1. The pump–probe instrument only had a single detection channel; hence, the TT and TR measurements were made consecutively. For these consecutive measurements, the TT and TR probe beams had to be realigned with the fiber optic cable connected to the detector. In addition, the measurements were carried out in two wavelength ranges, with an accompanying change of the detector (Si and InGaAs for visible and NIR range, respectively). Despite this, similar experimental conditions (the excitation energy and the studied spot of the sample) were ensured for each measurement (see Section S1 for further details).

**Figure 2:**
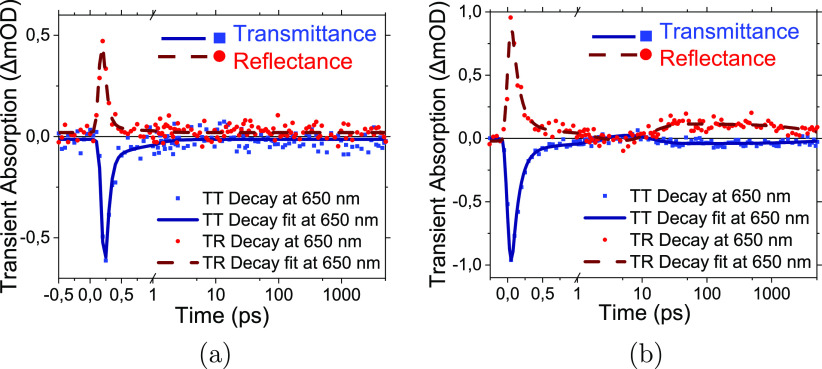
(a) TT and TR decay profiles of the multi-layer undoped graphene-based film (GTF) sample at 650 nm. (b) TT and TR decay profiles of multi-layer N-doped graphene-based films pyrolyzed at 1000 °C (NGF1000) at 650 nm. (The first picosecond is in linear, while the rest is in logarithmic scale.)

The transient transmittance (TT) and reflectance (TR) responses of GTF in Figure 2a were short-lived. This agrees with previous studies, which connected the fast relaxation with the fact that graphene is a zero band gap semiconductor.^25^ Immediately after excitation, the carriers are thermalized above the Dirac point by carrier–carrier scattering before rapid recombination and cooling to equilibrium temperature.^29^ The data in Figure 2a were fitted as a bi-exponential decay which delivered 0.1 and 0.58 ps time constants. Dawlaty et al.^25^ suggest that these two components result from carrier–carrier and carrier–phonon scattering, respectively, with the latter more affected by defect proportion. Similarly, optical pump–terahertz (THz) probe spectroscopy has shown biexponential decays due to coupling between optical phonons and hot carriers.^47,48^ Several publications suggest three decay components due to carrier–carrier,^29,30,49,50^ faster carrier–optical phonon, and slower carrier–acoustic phonon scattering, respectively. Since carrier–carrier scattering between 30 and 40 fs is beyond our instrument’s time resolution (100–200 fs), the last two components correspond to the fitted time constants. The TT and TR responses of graphite are also short-lived within the subpicosecond to picosecond range, with a response time constant showing a proportional dependence with the monitoring wavelength^26,27^ and a slightly increased lifetime because of increased out-of-plane motions in graphite.^51^

The TT and TR responses of one of the doped samples, NGF1000, are presented in Figure 2b (see Figures S7–S10, Section S4.2 for the full NGF series). Both the transmittance and reflectance responses have a fast decay (<1 ps) similar to multi-layer undoped graphene-based film (GTF), labeled as the “first wave”. In addition, there is a longer-lived response formed within 20–30 ps and only present for the multi-layer doped graphene-based films (NGF series), labeled as the “second wave”. The “second wave” is lower in magnitude than the “first wave” and more pronounced for TR than TT. Notably, in the time domain of the “second wave” (>10 ps), all the N-doped samples had this feature, while the undoped samples lacked this response.

The excitation density dependence of the TA response was checked, and a linear relation between the response intensity and excitation density was confirmed for both the “first” and “second waves” (see Figure S14, Section S4.3). This suggests a so-called linear regime and excludes multi-photon phenomena. In addition, a few excitation wavelengths (i.e., 320, 500, and 640 nm) were checked, and no essential wavelength dependence was observed (see Figure S15, Section S4.3).

**Figure 3:**
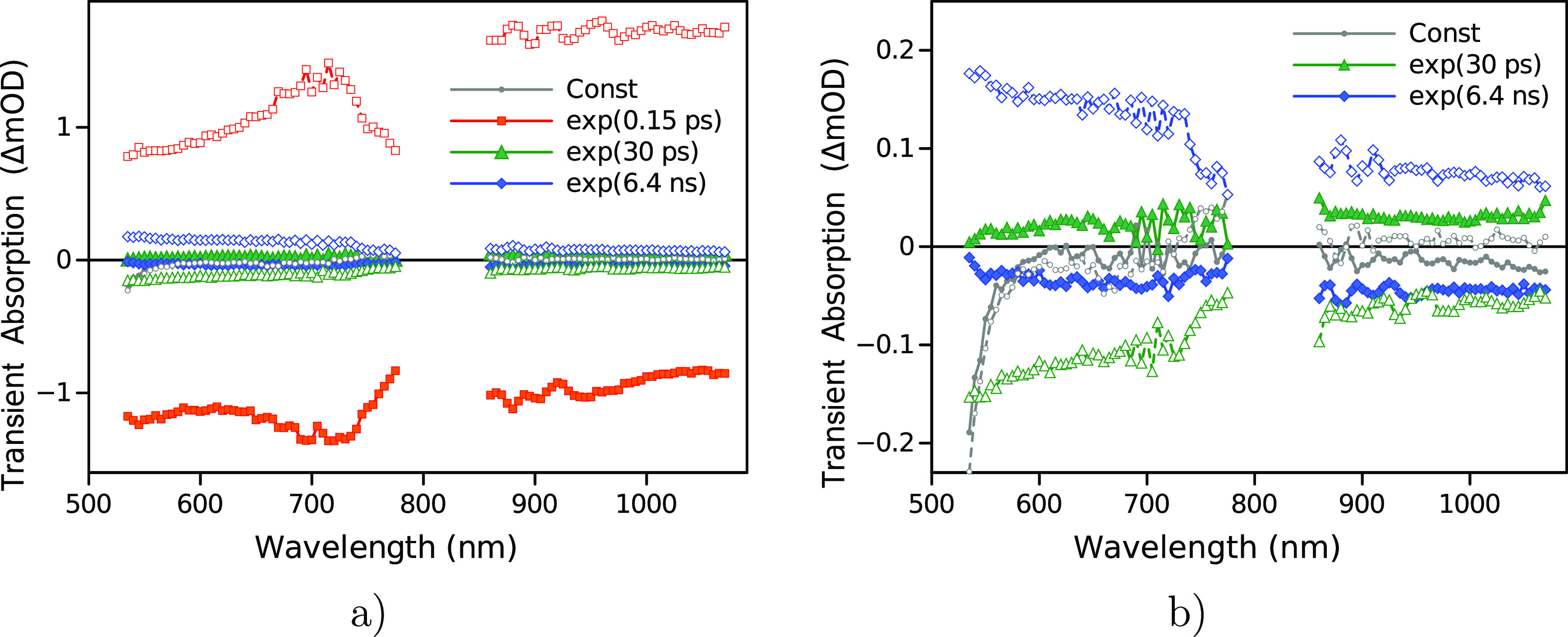
DAS resulting from the global fit of the TT and TR spectra for the NGF1000 sample. The transmittance and reflectance DAS are indicated by filled symbols and open symbols, respectively. Plots (a,b) represent the same spectra, but in plot (b), the scale is magnified to highlight the “second wave”.

The TT and TR data measured in the wavelength range 530–1100 nm were fitted globally using a three-exponential model to obtain the rough estimation of the time constants for the “first wave” decay (<1 ps), the formation of the “second wave” (20–30 ps), and its subsequent decay (>1 ns). It should be noted that the obtained time constants are common to both the TT and TR responses throughout the whole measured spectrum range as shown in Figure S16. In addition to the time constants, the fit provides the decay-associated spectra (DAS) or the spectra of the pre-exponential factors, *a*_*i*_(λ) in the fit model, , where *a*_0_(λ) is the disturbance of the detected probe independent of the delay time, e.g., scattered light from the pump. As an example, the obtained DAS for the NGF1000 sample are presented in Figure 3. The spectrum of *a*_0_(λ) is shown in the figure as “Const”, and it is virtually zero at all wavelengths except the blue side approaching the excitation at 500 nm (due to scattering of light from the pump). The DAS of other samples in the NGF series are shown in Figures S11–S13 (See Section S4.2). The comparison between the DAS of the “first wave” and the “second wave” at different pyrolysis temperatures is shown in Figures S17 and 18 (Section S4.4).

The mirror-like △*A*_T_(λ, *t*) and △*A*_R_(λ, *t*) responses (Figures 2 and 3) point to a stronger effect of the refractive index change, △*n*, than the absorption coefficient change, △*k*, on the measured signals. A decrease in △*n* upon photo-excitation decreases the reflected light and increases the transmitted light intensity, leading to mirror-like TT and TR responses. On the contrary, the change in the sample absorption must result in simultaneous increase (if △*k* < 0) or decrease (if △*k* > 0) of both TT and TR responses. However, the interpretation of the spectra is complicated as both *n* and *k* may change, and the effect of the change depends on the initial values of *n* and *k*.

The complex refractive index, *ñ* = *n* + *ik*, is determined by the complex dielectric function, , as was noted above. Therefore, the response can also be discussed in terms of changing the real, ε_1_(λ), and imaginary parts, ε_2_(λ), of the dielectric function, ε(λ) = ε_1_(λ) + *i*ε_2_(λ). Formally, , or *n*^2^ – *k*^2^ = ε_1_ and 2*nk* = ε_2_, meaning that *ñ* can be recalculated to ε and vice versa. The analysis of the dielectric function and its photo-induced change can be achieved using the D–L model, which provides information on the photophysical basis behind the signal changes.

The knowledge of the sample dielectric function, eq 1, can be used to predict the TT and TR responses to a small change of ε_1_ and ε_2_. The D–L model allows us to establish the linear relation between △*A*_T_ and △*A*_R_ and △ε_1_ and △ε_2_, provided that the change is small (the first-order approximation). In other words, a pair of measurements, △*A*_T_(λ, *t*) and △*A*_R_(λ, *t*), can be recalculated to a pair of △ε_1_(λ, *t*) and △ε_2_(λ, *t*), for a known dispersion model, eq 1. The model is available from the fits of the steady-state transmittance and reflectance spectra, as discussed above. The recalculations can be made for each measured wavelength (independent of delay time), subsequently providing an alternative interpretation of the same measured results. However, instead of recalculating the whole data array (△*A*_T_(λ, *t*), △*A*_R_(λ, *t*)) to another data array △ε(λ, *t*), the focus is shifted to the spectra of the “first” and “second waves”.

**Figure 4:**
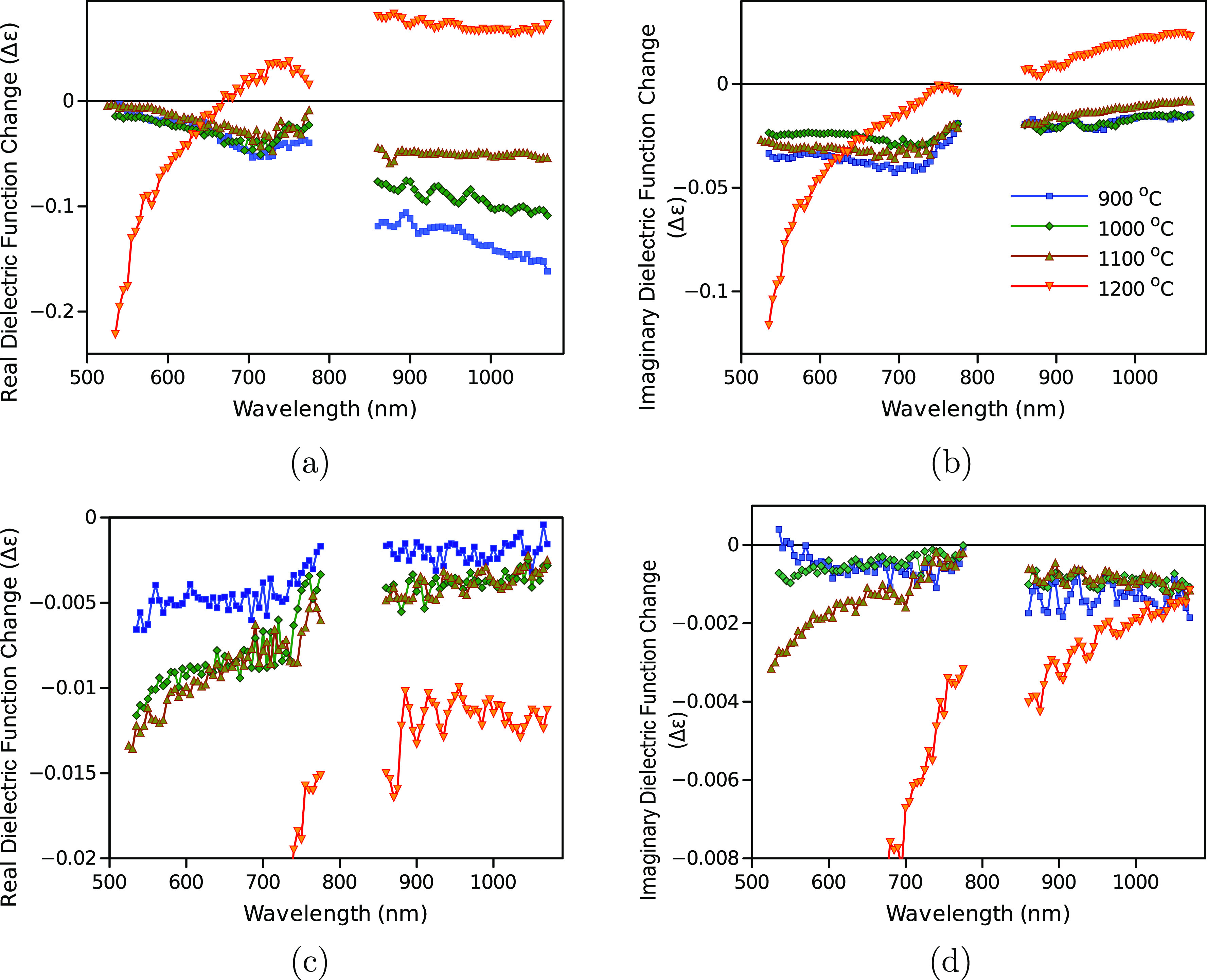
Spectra of real (a) and imaginary parts (b) of the dielectric function change of the “first wave” and the real (c) and imaginary (d) parts of the “second waves”. Pyrolysis temperatures are 900, 1000, 1100, and 1200 °C; the symbol codes and colors are indicated in plot (b). The △ε scales in plots (c,d) are magnified from Figure S19 to better highlight the spectra of samples pyrolyzed at lower temperatures.

The “first wave” spectra are calculated as the sum of all DAS obtained for TT and TR measurements, denoted previously as △*A*_T_ and △*A*_R_, respectively. This is justified by using zero time in the sum of exponentials, , where *a*_*i*_(λ) are the DAS. In practice, the fast component (0.15 ps in Figure 3) has a greater signal intensity than those of the two longer-lived components together; thus, the spectrum calculated as the sum of exponentials is almost the same as the fast component DAS. The spectrum of the longest-lived component (6.4 ns in Figure 3) is denoted as the “second wave”, which is justified by the fact that τ_3_ ≫ τ_2_ ≫ τ_1_. The calculated dielectric function spectra for the “first” and “second wave” are presented in Figure 4 for samples pyrolyzed at different temperatures.

The △ε(λ) responses of NGF900–1100 samples are reasonably similar, but the response of NGF1200 differs significantly for both the “first wave” and the “second wave”. In addition, the features of the △ε(λ) spectra for the “first” and the “second waves” are rather distinct from each other. In particular, the intensity of the real part is increasing toward the longer wavelengths for the “first wave” and decreasing toward the longer wavelengths for the “second wave”.

In order to determine the origin of the “first” and the “second waves”, the established D–L dielectric function model was used to predict the effect of variation of each individual D–L parameter on the TT and TR responses. This was achieved by adding a 1% disturbance to each individual parameter and calculating the resulting difference relative to the non-disturbed ground-state spectra △*A*_T_(λ) and △*A*_R_(λ) of the TT and TR measurements. The calculated responses are shown in Figure S20 (Section S4.5). From the figure, a change in Drude parameters, *E*_D_ or Γ_D_, has a stronger effect on the TT and TR responses in the red part of the spectrum, whereas the change in Lorentz parameters *A*_L_ or *E*_L_ has a stronger influence on the TT response toward the 530 nm side of the measured range of 530–1100 nm and gives almost a flat TR spectral response. Thus, at a qualitative level, the photo-induced change of dielectric function associated with the “first wave” (Figure 4a,b) is attributed to a change in the Drude component (free carriers or plasmon response) predominantly, namely, a photo-induced decrease of the Drude component. With the same logic, the “second wave” (Figure 4c,d) is conversely associated with a change in the Lorentz component.

Since the spectral features of the TT and TR responses are distinct for each of the D–L parameters (see Figure S20), the △ε(λ) spectra can be decomposed to distinct spectral responses associated with the photo-induced changes of the individual D–L parameters, or the △ε(λ) spectra are fitted to the change of the individual D–L parameters. Altogether, the D–L model depends on six parameters: ε_∞_, *E*_D_, Γ_D_, *A*_L_, *E*_L_, and Γ_L_. Therefore, two spectra, Re(△ε(λ)) = △ε_1_(λ) and Im(△ε(λ)) = △ε_2_(λ), can be fitted by tuning six parameter values: △ε_∞_, △*E*_D_, △Γ_D_, △*A*_L_, △*E*_L_, and △Γ_L_.

**Figure 5:**
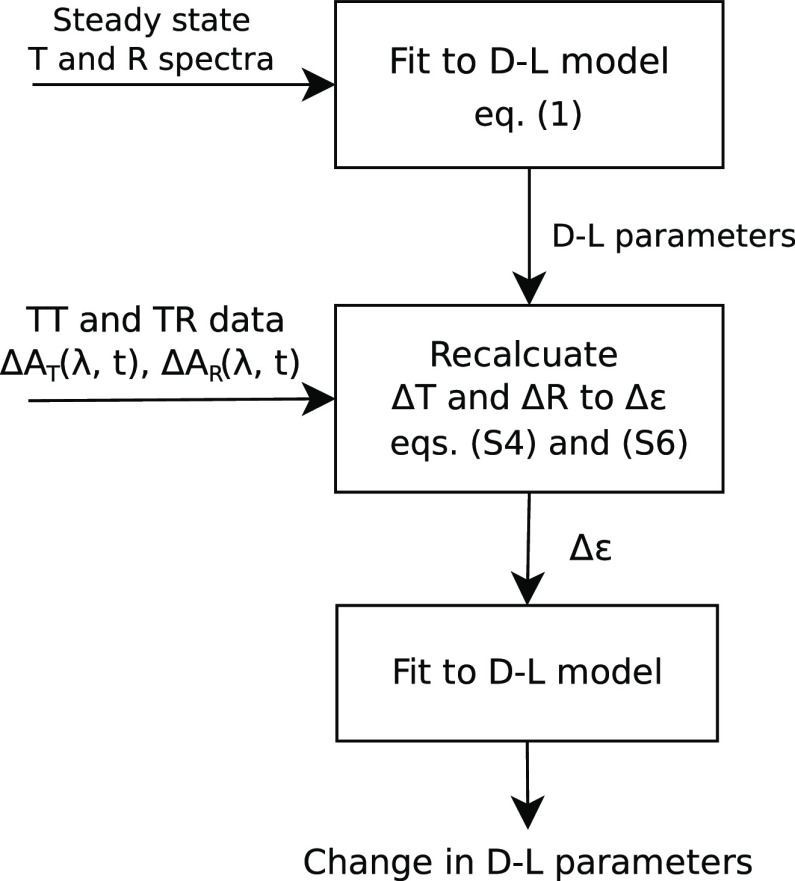
Schematic presentation of the steps involved in the spectral data analysis of the samples. (Supporting Information for eqs S4 and S6.)

The overall analysis procedure is divided in three steps as presented in Figure 5. First, the steady-state transmittance and reflectance spectra (Figure 1) are used to establish a suitable D–L model and determine the D–L parameters: ε_∞_, *E*_D_, Γ_D_, *A*_L_, *E*_L_, and Γ_L_ (Table 2). Second, the TT and TR measurements are fitted to obtain the DAS spectra (Figures 3, S11–S13) and to evaluate the TT and TR spectra of the “first” and “second waves”, which are recalculated to the corresponding △ε_1_(λ) and △ε_2_(λ) spectra using the D–L model (Figure 4). Finally, the △ε_1_(λ) and △ε_2_(λ) spectra are fitted to evaluate the contribution of the photo-induced disturbance of D–L parameters to the measured responses of the samples. The details of the calculations and fits are outlined in Section S4.1 of the Supporting Information.

**Figure 6:**
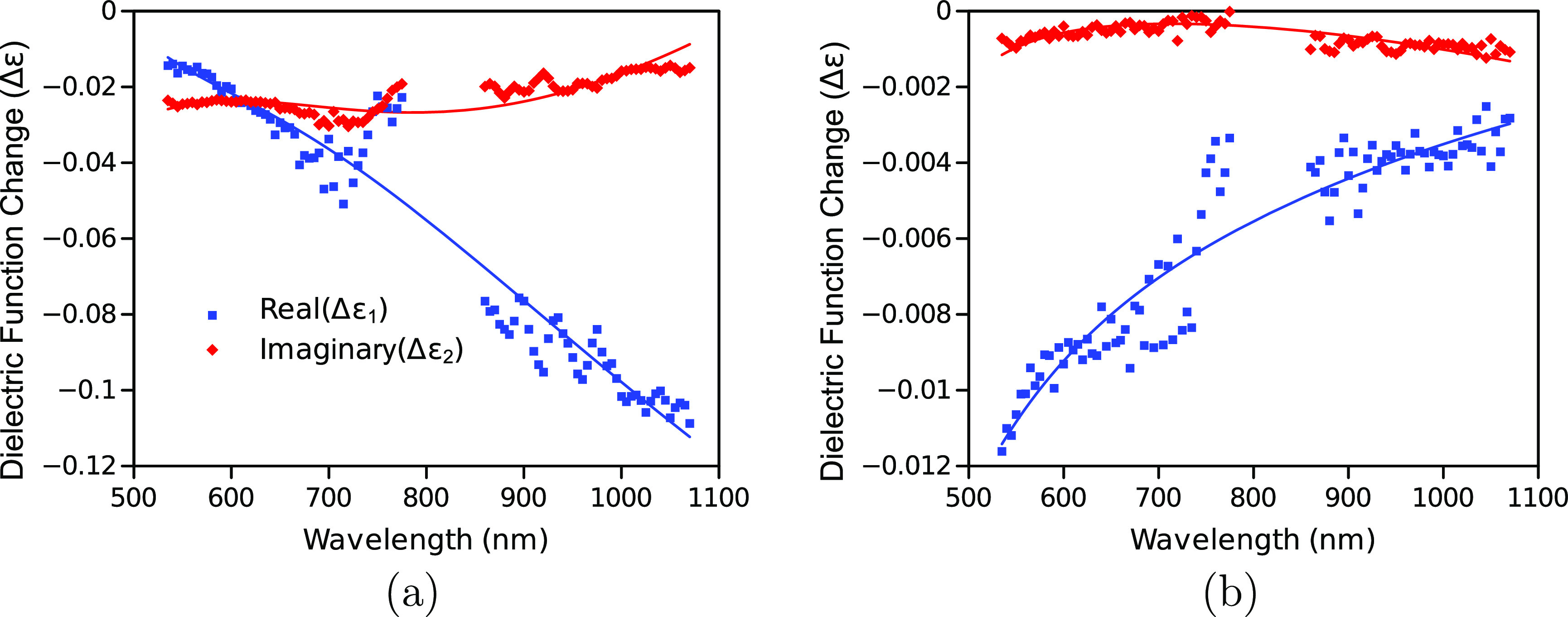
Fits of the real (blue) and imaginary (red) parts of the dielectric function change for the “first wave” (a) and the “second wave” (b) of the NGF1000 sample (the symbols are from the raw measured TT and TR responses, and the lines are from the fitting results).

**Figure 7:**
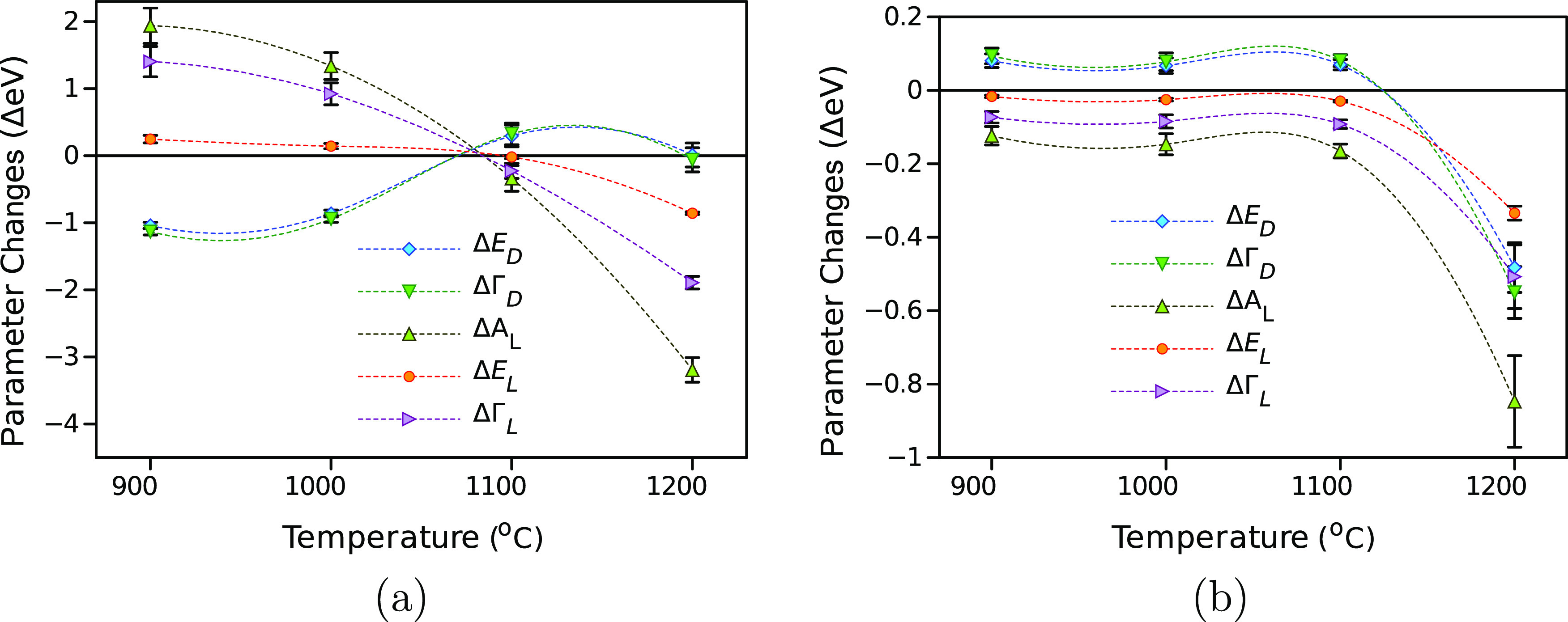
Fitted changes in the D–L parameters as a function of pyrolysis temperature for the “first wave” (a) and the “second wave” (b).

Figure 6 shows the fitting of NGF1000 as an example (see Figures S21–S23, Section S4.6 for other NGFs). The fittings of the samples pyrolyzed at 900–1100 °C fall within a reasonable accuracy compared to the measurements. Figure 7 summarizes the change in the D–L parameters of the multi-layer N-doped graphene-based film series associated with the “first” and “second waves”, respectively. The △ε_∞_ value is virtually zero for NGF900–NGF1100 samples but rises up by 1.3 for the “first wave” and by 0.45 for the “second wave” of the NGF1200 sample.

Interestingly, the fitted D–L parameters for the “first wave” of NGF1100 are very different from those of the NGF900 and NGF1000 samples, though their corresponding responses (Figure 4a,b) do not change radically. Conversely, the fitted D–L parameters for the “second wave” of the NGF1100 sample are similar to those of the NGF900 and NGF1000 samples, and the sharp change happens with the NGF1200 sample.

The “first wave” represents the instant change in the multi-layer graphene-based film optical properties at the event of photo-excitation. Although the largest absolute change associated with the “first wave” is obtained for *A*_L_, the static value of *A*_L_ is also the largest (Table 2), which means the overall contribution of △*A*_L_ is not the strongest. On the other hand, the largest relative change for the “first wave” was calculated for the Drude damping Γ_D_, which is inversely proportional to carrier scattering time or the plasma relaxation time.^44^ Therefore, as △Γ_D_ < 0, the photoexcitation increases the scattering time or conductivity at higher pyrolysis temperatures. At the same time, the Drude energy, *E*_D_, also decreases, which can be interpreted as a decrease in the number of “plasmon” electrons. This can be interpreted as the effect of “hot” electron generation, which differs significantly from the “ground state plasmons”. This is in agreement with photoelectron spectroscopy studies of similar n-doped graphene films,^29^ and graphene conductivity changes measured by THz spectroscopy, which were considered using the Drude model position of the Fermi level relative to the Dirac point.^47,52^ The overall explanation for this trend in Figure 7 is that the extra carriers in the π* band before excitation reduce the possible excited carriers owing to Pauli blocking,^29,47^ i.e., there is reduced available phase space and conductivity with increased N doping (lower pyrolysis temperatures).

In addition, the position of the Lorentz band, *E*_L_, virtually does not change, whereas the band width, Γ_L_, increases with higher pyrolysis temperatures. The changes associated with the Lorentz component are interpreted as an increase in intensity (△*A*_L_ > 0) and a broadening of the band (△Γ_L_ > 0) on event of the photo-excitation. A qualitative interpretation is that immediately after the excitation, there are more carriers available for the π → π* transitions (△*A*_L_ > 0), and there is a disturbance of the transition energies or the bandwidth broadening.

The D–L parameter changes associated with the “second wave” were an order of magnitude smaller and inverted (opposite sign of the change) compared to that in the “first wave”. The change in Γ_D_ (△Γ_D_ > 0) suggests a fall in conductivity compared to the ground state, while the *A*_L_ and Γ_L_ changes suggest a less efficient and narrower π → π* transition. This inversion in the parameters suggests that a different phenomenon opposite to opto-electronic contributions of the “first wave” starts to occur, with the carriers experiencing a restrictive force akin to trapping. It is interesting to notice that virtually no signal is observed in the time interval of 1–5 ps, though the later formation of the “second wave” (10–40 ps) clearly shows that there is no complete relaxation of the photo-generated carriers. Presumably, there is an intermediate state, which leaves no spectral features in the sample optical response within the 530–1100 nm monitoring range and which is a precursor to fill the N-doping centers. An interesting monitoring range would be the Lorentz band in the UV region, which is also known as Fano band. Unfortunately, the generation of probe light in this range requires specific accessories not available in our pump–probe instrument. However, Oum et al. reported a long-lived TA response in this range using a pump–supercontinuum probe technique.^30^ They reported a shift of the Fano band with recovery time extended to hundreds of picoseconds. This shift was attributed to the lattice heating. However, we observed the “second wave” only in the doped samples, which excludes heating as the main reason for the “second wave” as the heating effect must be the same for all samples. Consequently, there must be another mechanism to activate the doping centers, which determines the photo-catalytic activity of the multi-layer doped graphene-based film samples.

The reason to associate photo-catalytic activity of the multi-layered doped graphene-based film samples with the “second wave” is the lifetime, the signal virtually not degrading within our measurement scale. The doping centers were undetected in steady-state spectra due to the low concentration (2–3% N), as summarized in Table 1. The possible N-doping centers include pyridinic or quaternary N,^53,54^ as outlined in Table S1. However, it can be noticed that the most probable time scale for the catalytic reaction is much longer that available in our pump–probe instrument, 6 ns. Also, the measurements reported here were carried out in open air, while catalytic reactions require very different environments.

## Conclusions

This transient absorption study provides novel photophysical evidence of the unique behavior of multi-layer N-doped graphene-based films, derived from biomass waste compared to that of multi-layer pristine graphene-based films through a long-lived photoresponse from active N-doping centers. The steady-state and time-resolved spectra were analyzed within the Drude–Lorentz model and coupled with XPS data, showing that the “first wave” changes with increased pyrolysis temperatures (decreased N-doping) were due to increased conductivity, while those in the “second wave” were due to trapping of the photo-generated carriers. Since an additional Lorentz component (due to insufficient data on energy and the damping factor) was unutilized in our model, these results are considered only as indirect proof of the enhanced photo (electro) catalytic activity of the multi-layer N-doped graphene-based film samples due to N-doping centers.

## Supporting Information Available

Experimental Section (with full details of sample synthesis and characterization methods), additional acquired transient spectra (and decays) for the NGF series samples, and additional details, dielectric function spectra and parameter fits using the Drude–Lorentz model, and details of the python programs used for our fits (PDF)

## References

[ref1] Huang X.; Yin Z.; Wu S.; Qi X.; He Q.; Zhang Q.; Yan Q.; Boey F.; Zhang H. Graphene-Based Materials: Synthesis, Characterization, Properties, and Applications. Small 2011, 7, 1876–1902. 10.1002/smll.201002009. 21630440

[ref2] Novoselov K. S.; Geim A. K.; Morozov S. V.; Jiang D.; Zhang Y.; Dubonos S. V.; Grigorieva I. V.; Firsov A. A. Electric Field Effect in Atomically Thin Carbon Films. Science 2004, 306, 666–669. 10.1126/science.1102896. 15499015

[ref3] Farjadian F.; Abbaspour S.; Sadatlu M. A. A.; Mirkiani S.; Ghasemi A.; Hoseini-Ghahfarokhi M.; Mozaffari N.; Karimi M.; Hamblin M. R. Recent Developments in Graphene and Graphene Oxide: Properties, Synthesis, and Modifications: A Review. ChemistrySelect 2020, 5, 10200–10219. 10.1002/slct.202002501.

[ref4] Geim A. K.; Novoselov K. S. The Rise of Graphene. Nat. Mater. 2007, 6, 183–191. 10.1038/nmat1849. 17330084

[ref5] Das S.; Choi W.. In Graphene: Synthesis and Applications; Choi W., Lee J.-W., Eds.; CRC Press: Boca Raton, 2011; Chapter 2, pp 27–63.

[ref6] Wick P.; Louw-Gaume A. E.; Kucki M.; Krug H. F.; Kostarelos K.; Fadeel B.; Dawson K. A.; Salvati A.; Vázquez E.; Ballerini L.; et al. Classification Framework for Graphene-Based Materials. Angew. Chem., Int. Ed. 2014, 53, 7714–7718. 10.1002/anie.201403335. 24917379

[ref7] Bianco A.; Cheng H.-M.; Enoki T.; Gogotsi Y.; Hurt R. H.; Koratkar N.; Kyotani T.; Monthioux M.; Park C. R.; Tascon J. M.; Zhang J.; Zhang J. All in the Graphene Family – A Recommended Nomenclature for Two-Dimensional Carbon Materials. Carbon 2013, 65, 1–6. 10.1016/j.carbon.2013.08.038.

[ref8] Zhou X.; Zhao C.; Wu G.; Chen J.; Li Y. DFT Study on the Electronic Structure and Optical Properties of N, Al, and N-Al Doped Graphene. Appl. Surf. Sci. 2018, 459, 354–362. 10.1016/j.apsusc.2018.08.015.

[ref9] Laref A.; Ahmed A.; Bin-Omran S.; Luo S. J. First-Principle Analysis of the Electronic and Optical Properties of Boron and Nitrogen Doped Carbon Mono-Layer Graphenes. Carbon 2015, 81, 179–192. 10.1016/j.carbon.2014.09.047.

[ref10] Orlita M.; Potemski M. Dirac Electronic States in Graphene Systems: Optical Spectroscopy Studies. Semicond. Sci. Technol. 2010, 25, 063001 10.1088/0268-1242/25/6/063001.

[ref11] Antonietti M.; Lopez-Salas N.; Primo A. Adjusting the Structure and Electronic Properties of Carbons for Metal-Free Carbocatalysis of Organic Transformations. Adv. Mater. 2019, 31, 1805719 10.1002/adma.201805719. 30561777

[ref12] Baldoví H. G.; Albarracín F.; Álvaro M.; Ferrer B.; García H. Influence of Dopant Loading on the Photo- and Electrochemical Properties of (N, O)-Co-doped Graphene. ChemPhysChem 2015, 16, 2094–2098. 10.1002/cphc.201500306. 25968612

[ref13] Shih P.-H.; Do T.-N.; Gumbs G.; Lin M.-F. Electronic and Optical Properties of Doped Graphene. Phys. E 2020, 118, 113894 10.1016/j.physe.2019.113894.

[ref14] Olaniyan O.; Maphasha R.; Madito M.; Khaleed A.; Igumbor E.; Manyala N. A Systematic Study of the Stability, Electronic and Optical Properties of Beryllium and Nitrogen Co-Doped Graphene. Carbon 2018, 129, 207–227. 10.1016/j.carbon.2017.12.014.

[ref15] Goudarzi M.; Parhizgar S.; Beheshtian J. Electronic and Optical Properties of Vacancy and B, N, O and F Doped Graphene: DFT Study. Opto-Electron. Rev. 2019, 27, 130–136. 10.1016/j.opelre.2019.05.002.

[ref16] Latorre-Sánchez M.; Primo A.; García H. P-Doped Graphene Obtained by Pyrolysis of Modified Alginate as a Photocatalyst for Hydrogen Generation from Water–Methanol Mixtures. Angew. Chem., Int. Ed. 2013, 52, 11813–11816. 10.1002/anie.201304505. 24105902

[ref17] Navalón S.; Herance J. R.; Álvaro M.; García H. General Aspects in the Use of Graphenes in Catalysis. Mater. Horiz. 2018, 5, 363–378. 10.1039/C8MH00066B.

[ref18] Duan J.; Chen S.; Jaroniec M.; Qiao S. Z. Heteroatom-Doped Graphene-Based Materials for Energy-Relevant Electrocatalytic Processes. ACS Catal. 2015, 5, 5207–5234. 10.1021/acscatal.5b00991.

[ref19] Kumar R.; Sahoo S.; Joanni E.; Singh R. K.; Maegawa K.; Tan W. K.; Kawamura G.; Kar K. K.; Matsuda A. Heteroatom Doped Graphene Engineering for Energy Storage and Conversion. Mater. Today 2020, 39, 47–65. 10.1016/j.mattod.2020.04.010.

[ref20] Albero J.; Mateo D.; García H. Graphene-Based Materials as Efficient Photocatalysts for Water Splitting. Molecules 2019, 24, 906 10.3390/molecules24050906. 30841539 PMC6429481

[ref21] Putri L. K.; Ng B.-J.; Ong W.-J.; Lee H. W.; Chang W. S.; Chai S.-P. Heteroatom Nitrogen- and Boron-Doping as a Facile Strategy to Improve Photocatalytic Activity of Standalone Reduced Graphene Oxide in Hydrogen Evolution. ACS Appl. Mater. Interfaces 2017, 9, 4558–4569. 10.1021/acsami.6b12060. 28068056

[ref22] Putri L. K.; Ong W.-J.; Chang W. S.; Chai S.-P. Heteroatom Doped Graphene in Photocatalysis: A Review. Appl. Surf. Sci. 2015, 358, 2–14. 10.1016/j.apsusc.2015.08.177.

[ref23] Bie C.; Yu H.; Cheng B.; Ho W.; Fan J.; Yu J. Design, Fabrication, and Mechanism of Nitrogen-Doped Graphene-Based Photocatalyst. Adv. Mater. 2021, 33, 2003521 10.1002/adma.202003521. 33458902

[ref24] Pogna E. A. A.; Jia X.; Principi A.; Block A.; Banszerus L.; Zhang J.; Liu X.; Sohier T.; Forti S.; Soundarapandian K.; et al. Hot-Carrier Cooling in High-Quality Graphene Is Intrinsically Limited by Optical Phonons. ACS Nano 2021, 15, 11285–11295. 10.1021/acsnano.0c10864. 34139125 PMC8320233

[ref25] Dawlaty J. M.; Shivaraman S.; Chandrashekhar M.; Rana F.; Spencer M. G. Measurement of Ultrafast Carrier Dynamics in Epitaxial Graphene. Appl. Phys. Lett. 2008, 92, 042116 10.1063/1.2837539. 19367881

[ref26] Breusing M.; Ropers C.; Elsaesser T. Ultrafast Carrier Dynamics in Graphite. Phys. Rev. Lett. 2009, 102, 086809 10.1103/PhysRevLett.102.086809. 19257774

[ref27] Seibert K.; Cho G. C.; Kütt W.; Kurz H.; Reitze D. H.; Dadap J. I.; Ahn H.; Downer M. C.; Malvezzi A. M. Femtosecond Carrier Dynamics in Graphite. Phys. Rev. B 1990, 42, 2842–2851. 10.1103/PhysRevB.42.2842. 9995773

[ref28] Kadi F.; Winzer T.; Knorr A.; Malic E. Impact of Doping on the Carrier Dynamics in Graphene. Sci. Rep. 2015, 5, 16841 10.1038/srep16841. 26577536 PMC4649495

[ref29] Johannsen J. C.; Ulstrup S.; Crepaldi A.; Cilento F.; Zacchigna M.; Miwa J. A.; Cacho C.; Chapman R. T.; Springate E.; Fromm F.; et al. Tunable Carrier Multiplication and Cooling in Graphene. Nano Lett. 2015, 15, 326–331. 10.1021/nl503614v. 25458168

[ref30] Oum K.; Lenzer T.; Scholz M.; Jung D. Y.; Sul O.; Cho B. J.; Lange J.; Muller A. Observation of Ultrafast Carrier Dynamics and Phonon Relaxation of Graphene from the Deep-Ultraviolet to the Visible Region. J. Phys. Chem. C 2014, 118, 6454–6461. 10.1021/jp4072197.

[ref31] Primo A.; Atienzar P.; Sanchez E.; Delgado J. M.; García H. From Biomass Wastes to Large-Area, High-Quality, N-Doped Graphene: Catalyst-Free Carbonization of Chitosan Coatings on Arbitrary Substrates. Chem. Commun. 2012, 48, 9254–9256. 10.1039/C2CC34978G. 22875403

[ref32] Sun D.; Wu Z.-K.; Divin C.; Li X.; Berger C.; Heer W. A. d.; First P. N.; Norris T. B. Ultrafast Dynamics and Interlayer Thermal Coupling of Hot Carriers in Epitaxial Graphene. Phys. Status Solidi C 2009, 6, 470–473. 10.1002/pssc.200880359.

[ref33] He J.; Anouar A.; Primo A.; García H. Quality Improvement of Few-Layers Defective Graphene from Biomass and Application for H_2_ Generation. Nanomaterials 2019, 9, 895 10.3390/nano9060895. 31248147 PMC6632024

[ref34] Dovbeshko G. I.; Romanyuk V. R.; Pidgirnyi D. V.; Cherepanov V. V.; Andreev E. O.; Levin V. M.; Kuzhir P. P.; Kaplas T.; Svirko Y. P. Optical Properties of Pyrolytic Carbon Films Versus Graphite and Graphene. Nanoscale Res. Lett. 2015, 10, 234 10.1186/s11671-015-0946-8. 26055479 PMC4456585

[ref35] Song B.; Gu H.; Zhu S.; Jiang H.; Chen X.; Zhang C.; Liu S. Broadband Optical Properties of Graphene and HOPG investigated by Spectroscopic Mueller Matrix Ellipsometry. Appl. Surf. Sci. 2018, 439, 1079–1087. 10.1016/j.apsusc.2018.01.051.

[ref36] Rendón-Patiño A.; Niu J.; Doménech-Carbó A.; García H.; Primo A. Polystyrene as Graphene Film and 3D Graphene Sponge Precursor. Nanomaterials 2019, 9, 101 10.3390/nano9010101. 30654444 PMC6358832

[ref37] Jerng S.-K.; Seong Yu D.; Hong Lee J.; Kim C.; Yoon S.; Chun S.-H. Graphitic Carbon Growth on Crystalline and Amorphous Oxide Substrates using Molecular Beam Epitaxy. Nanoscale Res. Lett. 2011, 6, 565 10.1186/1556-276X-6-565. 22029707 PMC3213075

[ref38] Shen C. C.; Lin C. T.; Li L. J.; Liu H. L. Charge Dynamics and Electronic Structures of Monolayer Graphene with Molecular Doping. Appl. Phys. Lett. 2012, 101, 111907 10.1063/1.4752131.

[ref39] Mohandoss M.; Nelleri A. Optical Properties of Sunlight Reduced Graphene Oxide using Spectroscopic Ellipsometry. Opt. Mater. 2018, 86, 126–132. 10.1016/j.optmat.2018.09.035.

[ref40] Chaudhuri K.; Alhabeb M.; Wang Z.; Shalaev V. M.; Gogotsi Y.; Boltasseva A. Highly Broadband Absorber Using Plasmonic Titanium Carbide (MXene). ACS Photonics 2018, 5, 1115–1122. 10.1021/acsphotonics.7b01439.

[ref41] Matsumoto T.; Koizumi T.; Kawakami Y.; Okamoto K.; Tomita M. Perfect Blackbody Radiation from a Graphene Nanostructure with Application to High-Temperature Spectral Emissivity Measurements. Opt. Express 2013, 21, 30964–30974. 10.1364/OE.21.030964. 24514669

[ref42] Radović I.; Borka D.; Mišković Z. L. Theoretical Modeling of Experimental HREEL Spectra for Supported Graphene. Phys. Lett. A 2014, 378, 2206–2210. 10.1016/j.physleta.2014.06.001.

[ref43] Ma H.; Liu X.; Gao C.; Yin Y. The Calculated Dielectric Function and Optical Properties of Bimetallic Alloy Nanoparticles. J. Phys. Chem. C 2020, 124, 2721–2727. 10.1021/acs.jpcc.9b11154.

[ref44] Andvaag I. R.; Morhart T. A.; Clarke O. J. R.; Burgess I. J. Hybrid Gold–Conductive Metal Oxide Films for Attenuated Total Reflectance Surface Enhanced Infrared Absorption Spectroscopy. ACS Appl. Nano Mater. 2019, 2, 1274–1284. 10.1021/acsanm.8b02155.

[ref45] Katsidis C. C.; Siapkas D. I. General Transfer-Matrix Method for Optical Multilayer Systems with Coherent, Partially Coherent, and Incoherent Interference. Appl. Opt. 2002, 41, 3978–3987. 10.1364/AO.41.003978. 12099609

[ref46] Byrnes S. J. Multilayer Optical Calculations. 2016; https://arxiv.org/abs/1603.02720, (accessed Jan 16, 2023).

[ref47] Tomadin A.; Hornett S. M.; Wang H. I.; Alexeev E. M.; Candini A.; Coletti C.; Turchinovich D.; Kläui M.; Bonn M.; Koppens F. H. L.; et al. The Ultrafast Dynamics and Conductivity of Photoexcited Graphene at Different Fermi Energies. Sci. Adv. 2018, 4, eaar5313 10.1126/sciadv.aar5313. 29756035 PMC5947979

[ref48] Jensen S. A.; Mics Z.; Ivanov I.; Varol H. S.; Turchinovich D.; Koppens F. H. L.; Bonn M.; Tielrooij K. J. Competing Ultrafast Energy Relaxation Pathways in Photoexcited Graphene. Nano Lett. 2014, 14, 5839–5845. 10.1021/nl502740g. 25247639

[ref49] Wang H.; Strait J. H.; George P. A.; Shivaraman S.; Shields V. B.; Chandrashekhar M.; Hwang J.; Rana F.; Spencer M. G.; Ruiz-Vargas C. S.; et al. Ultrafast Relaxation Dynamics of Hot Optical Phonons in Graphene. Appl. Phys. Lett. 2010, 96, 081917 10.1063/1.3291615.

[ref50] Shang J.; Luo Z.; Cong C.; Lin J.; Yu T.; Gurzadyan G. G. Femtosecond UV-Pump/Visible-Probe Measurements of Carrier Dynamics in Stacked Graphene Films. Appl. Phys. Lett. 2010, 97, 163103 10.1063/1.3504704.

[ref51] Carbone F.; Aubock G.; Cannizzo A.; Van Mourik F.; Nair R.; Geim A.; Novoselov K.; Chergui M. Femtosecond Carrier Dynamics in Bulk Graphite and Graphene Paper. Chem. Phys. Lett. 2011, 504, 37–40. 10.1016/j.cplett.2011.01.052.

[ref52] Frenzel A. J.; Lui C. H.; Shin Y. C.; Kong J.; Gedik N. Semiconducting-to-Metallic Photoconductivity Crossover and Temperature-Dependent Drude Weight in Graphene. Phys. Rev. Lett. 2014, 113, 056602 10.1103/PhysRevLett.113.056602. 25126929

[ref53] Zhang Z.; Yu L.; Tu Y.; Chen R.; Wu L.; Zhu J.; Deng D. Unveiling the Active Site of Metal-Free Nitrogen-doped Carbon for Electrocatalytic Carbon Dioxide Reduction. Cell Rep. Phys. Sci. 2020, 1, 100145 10.1016/j.xcrp.2020.100145.

[ref54] Saidi W. Oxygen Reduction Electrocatalysis Using N-Doped Graphene Quantum-Dots. J. Phys. Chem. Lett. 2013, 4, 4160–4165. 10.1021/jz402090d.

