# Supplementary material for: Long-Lived Photo-Response of Multi-Layer N-Doped Graphene-Based Films
Source: J Phys Chem C Nanomater Interfaces. 2023 Aug 30;127(36):17896–905. doi: 10.1021/acs.jpcc.3c04670 (PMC10510389; doi:10.1021/acs.jpcc.3c04670)
Supplement: Supplementary file 1 — jp3c04670_si_001.pdf [file jp3c04670_si_001.pdf]

Supporting Information : Long–Lived Photo–Response of Multi–Layer N–Doped Graphene–Based Films

Jokotadeola A. Odutola,[†] Horaţiu Szalad,[‡] Josep Albero,[‡] Hermenegildo García,[‡] and Nikolai V. Tkachenko[*,†]

†*Photonics compound and Nanomaterials (Chemistry and Advanced Materials Group), Faculty of Engineering and Natural Sciences, Tampere University, Korkeakoulunkatu 8, FI-33720 Tampere, Finland.*

‡*Instituto Universitario de Tecnología Química, Universitat Politècnica de València, Avda. de los Naranjos s/n, 46022 Valencia, Spain.*

E-mail: nikolai.tkachenko@tuni.fi

Contents

S1 Experimental Section **S2**

S2 Sample Structure Characterization **S7**

S3 Steady State Spectroscopy **S11**

S3.1 Steady State Spectra Modeling S11

S3.2 Film Thickness Modeling S17

S4 Transient Absorption Spectroscopy **S17**

S4.1 Analysis of Transient Measurements S17

S4.2 Transient Absorption Measurements S20

S4.3 Excitation Density, Excitation Wavelength and Monitoring Wavelength Dependence S24

S4.4 Comparison of the Transient Absorption Response S26

S4.5 The Effect of D-L Parameter Changes on the Transient Absorption S29

S4.6 The Photo-Induced Change of the Dielectric Function S30

S5 Data Analysis Software **S31**

References **S32**

S1 Experimental Section

Sample Preparation. Low molecular weight chitosan (0.5 g) was dissolved in aqueous acidic solution (0.23 g of acetic acid in 25 mL of Miliq water). The solution was filtered through a syringe having a membrane filter of 0.45 μm diameter pore size to remove impurities present in commercial chitosan. The multi-layer defective graphene-based films were supported on pre-cleaned quartz plates (2×2 cm). The quartz substrates were sequentially

sonicated in Miliq water and acetone for 15 minutes. Then, dried under N_2 flow and O_2-plasma treated for 15 minutes before chitosan solution deposition. 300 μL of filtered chitosan solution were spin coated at 6 000 r.p.m. during 1 minute. Samples pyrolysis was carried out under argon (Ar) atmosphere in a tubular furnace at 5 °C/min rate up to 900, 1000, 1100 and 1200 °C and the temperature held for 2 h. The multi-layer graphene-based films were then cooled down to room temperature under Ar flow. For comparison purposes, multi-layer undoped graphene-based samples were prepared by polystyrene sublimation at 900 °C under Ar atmosphere.

Raman Spectroscopy Raman spectrum was collected with a Horiba Jobin Yvon-Labram HR UV-Visible-NIR (200–1 600 nm) Raman Microscope Spectrometer, using a laser with the wavelength of 632 nm. The spectrum was collected from 10 scans at a resolution of 2 cm^{-1}. XPS spectra were measured on a SPECS spectrometer equipped with a Phoibos 150 9MCD detector using a non-monochromatic X-ray source (Al and Mg) operating at 200 W. The samples were evacuated in the prechamber of the spectrometer at $1{\cdot}10^{-9}$ mbar. The measured intensity ratios of the components were obtained from the area of the corresponding peaks after nonlinear Shirley-type background subtraction and corrected by the transition function of the spectrometer.

X-Ray Photoelectron Spectroscopy All XPS spectra were recorded via a SPECS spectrometer (SPECS Surface Nano Analysis GmbH, Berlin, Germany) with a Phoibos 150 MCD-9 detector. The non-monochromatic X-Ray source (Al and Mg) was operated at 200 W. Before data acquisition, the XPS setup antechamber was evacuated at 10^{-9} mbar. The work function was calibrated with Ag, Au, and Cu standards, hence a value of 4.2440 eV was obtained. The intensity ratios of all analyzed samples were obtained after nonlinear Shirley-type background subtraction and correction by the transition function of the spectrometer.

Atomic Force Microscopy (AFM) measurements were conducted in the contact mode in air to measure thickness and roughness at ambient temperature using a Veeco AFM apparatus. The films were scratched to determine the thickness. It should be noted that AFM were not measured in a clean room and, therefore, films on quartz substrates may contain dust that will be detectable by these techniques.

Steady State Spectroscopy. The steady state transmittance, T, of the samples were measured with a Shimadzu UV-3600 series spectrophotometer. The specular reflectance, R, of the samples were measured with a specular reflectance attachment (for 5° incidence angle). The reflectance spectra are measured relative to ideal "100 %" reflecting mirror. We found that the supplied aluminium reference mirror has relatively high (more than 10 %) deviation from ideal reflectivity in the desired range of 200–1200 nm. Therefore, we used a quartz plate as the reference, for which the transmittance was measured in a standard way and we assumed that the reflectance is $R = 1 - T$. Thus corrected data were more reasonable, though the accuracy of reflectance measurements in the UV part (200–300 nm) was still poor.

The "real absorbance", A, was calculated from the specular reflectance, R, and transmittance, T, of the sample using equation

$$A = -\log \frac{T}{1-R} \tag{S1}$$

Transient Absorption Spectroscopy. The transient absorption measurements of the samples were carried out using a laser pump-probe set-up. The fundamental laser pulses at repetition rate 1 kHz and pulse width 100 fs were generated at 800 nm by the Libra F system, Coherent Inc., which was coupled with an optical parametric amplifier (OPA) Topas C, Light Conversion Ltd. These laser pulses were used to produce the pump beam to excite the sample and the probe beam (white continuum) to monitor the spectra. The pump beam wavelength at 500 nm excitation was generated by channeling a portion of the fundamental

laser to the OPA. The pump beam average power was set at 500 ± 50 µW (corresponding to the excitation density or roughly 0.1 $mJ\,cm^{-2}$) for the multi-layer N-doped and undoped graphene-based film samples.

For the probe beam, a sapphire crystal was used to produce a continuum of white light in the near-infrared (NIR) and visible ranges. The central detection wavelength in the NIR and visible ranges were 960 and 600 nm, respectively. The probe beam was passed through a delay line for the differential response in time. The transient absorption responses of the probe beam were measured using an ExciPro TA spectrometer (CDP, Inc.). The spectrometer was coupled with an InGaAs diode array to detect NIR wavelengths and a Si charge coupled device (CCD) array to detect visible range wavelengths. For the transient absorption measurements, both the transient transmittance (TT) and transient reflectance (TR) modes were explored, as schematically presented in Figure S1. All the beams (pump, probe transmitted and reflected) propagated to/from the sample surface at roughly normal incidence, with deviation from the normal $\alpha \leq 5$ degrees, therefore all the modeling and calculations were carried out assuming normal incidence.

The pump-probe instrument only had a single detection channel, hence, the TT and TR measurements were made consecutively. For these consecutive measurements, the TT and TR probe beams had to be realigned with the fibre optic cable connected to the detector. In addition, the measurements were carried in two wavelength ranges, with an accompanying change of the detector (Si or InGaAs for visible or NIR range, respectively). Despite this, similar experimental conditions (the excitation energy and the studied spot of the sample) were ensured for each measurement.

Independent of the measuring mode, TT or TR, the instrument recalculated the measured change in probe to the change of optical density $\triangle A$, which are referred to as $\triangle A_T$ or $\triangle A_R$, respectively. To analyse the data we used global exponential fit of both $\triangle A_T(\lambda, t)$ and $\triangle A_R(\lambda, t)$ simultaneously:

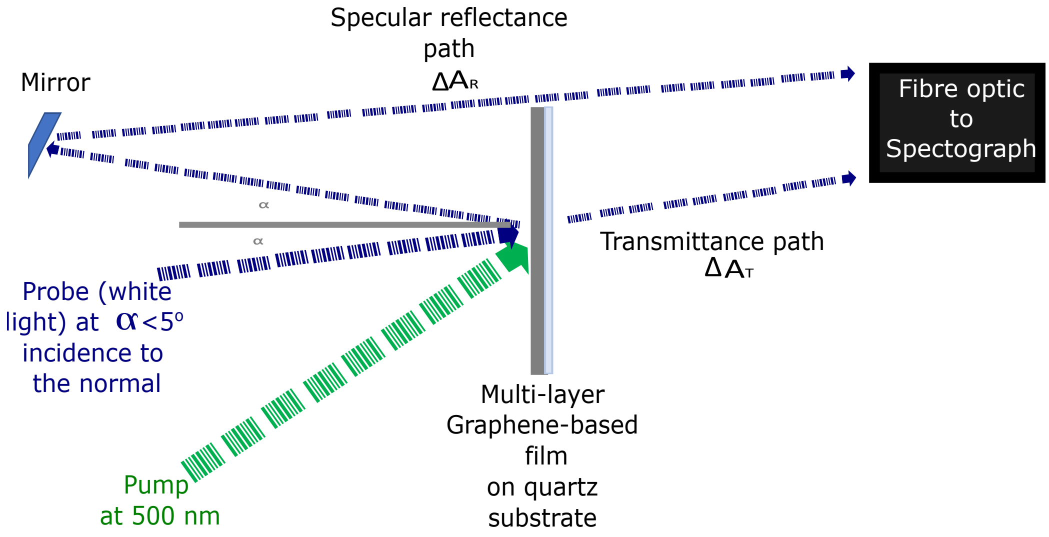

Figure S1: Schematic presentation of the TT and TR measurements with the transient absorption spectroscopy setup.

$$\triangle A_{(T/R)}(\lambda, t) = A_{(T/R)0}(\lambda) + \sum_{i=1}^{N} A_{(T/R)i}(\lambda) \exp(-t/\tau_i) \quad \text{(S2)}$$

where $\triangle A_{(T/R)}(\lambda, t)$ are the transient absorption (in transmittance, $\triangle A_T$, or reflectance, $\triangle A_R$, modes) at wavelength λ and delay time t, $A_{(T/R)0}(\lambda)$ are the responses independent of the delay time, e.g. scattered pump, $A_{(T/R)i}(\lambda)$ are the pre-exponential factors or decay associated spectra (DAS), τ_i are the time constants common to both transmittance and reflectance responses and associated with DAS of this two modes, and N is the number of exponential terms used ($N = 3$ in most cases). The in-house developed fit program uses convolution with the instrument response (100 fs) modeled by a Gaussian pulse and makes group velocity dispersion compensation in each measured wavelength range (530–770 nm and 860–1080 nm in this study) by the second order approximation of the dispersion curve. Typically, the estimated instrument response was 100–200 fs. The program, `ppfit.py`, is an open-source software and available on request.

S2 Sample Structure Characterization

The formation of multi-layer defective graphene-based films was confirmed by Raman spectroscopy as shown in Figure S2.

Further study of the chemical composition of the obtained films was carried out by X-ray Photoelectron Spectroscopy (XPS). The high resolution XPS C 1s, O 1s and N 1s spectra of the different samples is presented as Figure S3. All samples present similar components but the relative percentage of each one varies between them.

The C 1s peaks of all samples have been deconvoluted in 4 different components attributed to sp^2 C, C−O/C−N, C=O and O−C=O bonds. It can be noted that the C 1s band is well presented by a single Gaussian corresponding to sp^3 hybridization, and no sign of the sp^2 can be seen on the lower energy side of the band. It was reported that the ratio of sp^3 and sp^2 bands in graphite is roughly 1:9,[1] which could not be detected in our case. This

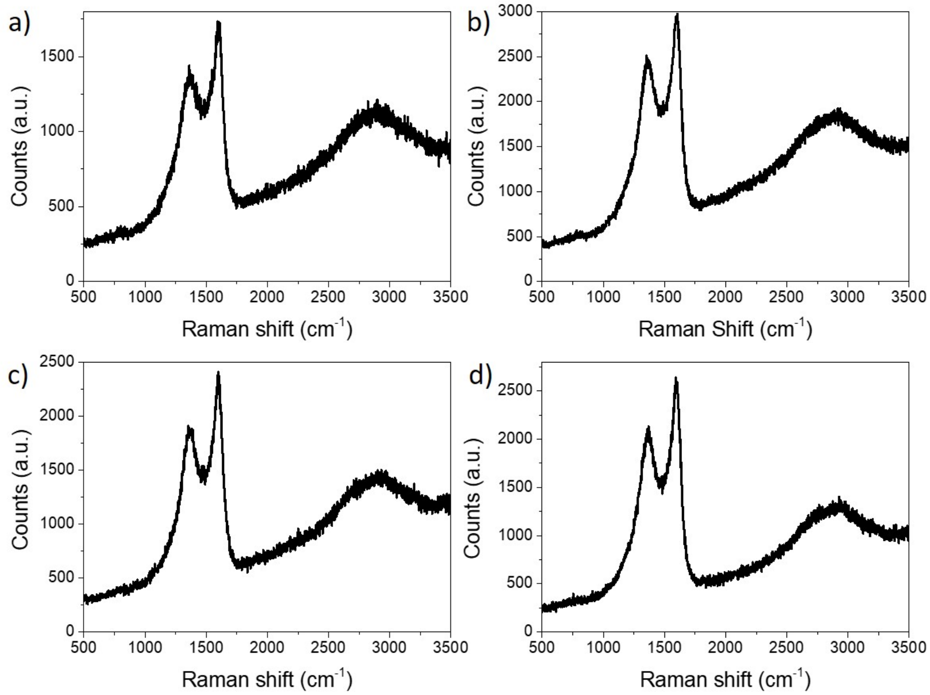

Figure S2: Raman spectra of the NGF900 (a), NGF1000 (b), NGF1100 (c) and NGF1200 (d) samples.

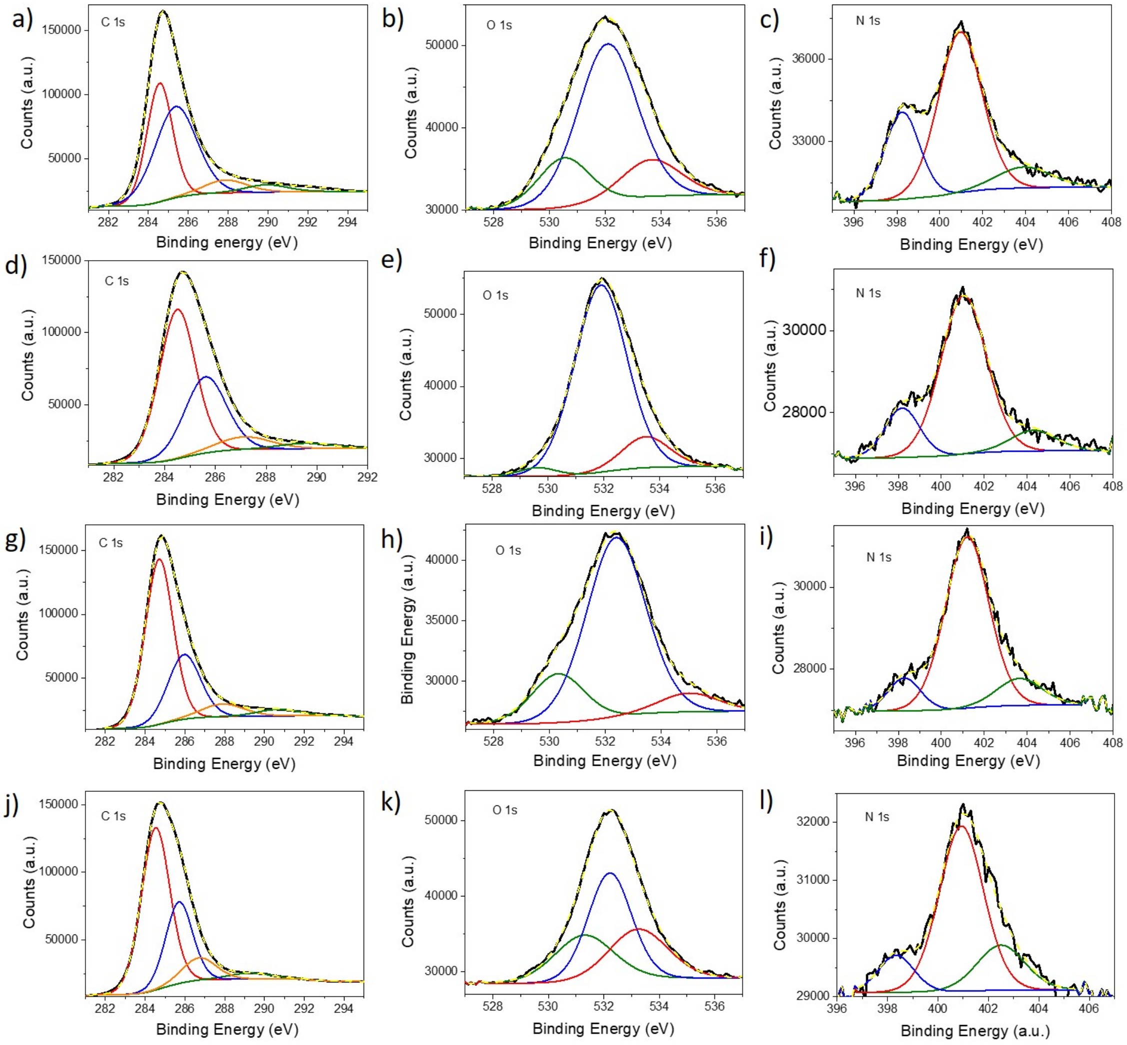

Figure S3: XPS peaks of the NGF900 (a-c), NGF1000 (d-f), NGF1100 (g-i) and NGF1200 (j-l) samples showing their experimental C 1s, O 1s and N 1s compositions and the best deconvolution to individual components.

result indicates that graphite type of structure is minor in our samples if any. The O 1s peaks have been deconvoluted in three components related to O−C, O=C, and O−C=O. Finally, the N 1s peaks present three components assigned to pyridinic-N, quaternary-N and N-oxides. Table S1 summarises the percentage of each component in the different samples. Alternatively, the C, N, and O content in each sample are also summarized in Table 1 (in the main text). The N content decreased with the pyrolysis temperature, while the C content increased. Conversely, the trend for the O content from the incomplete carbonization of the chitosan precursor did not show such a smooth transition, although there was an overall decrease from NGF900 to NGF1100.

Table S1: Summary of the percentages of the components in C 1s, O 1s and N 1s peaks of the different samples.

	Components	NGF900	NGF1000	NGF1100	NGF1200
C 1s	sp^2 (284.50 eV)	40.87%	56.69%	61.30%	59.05%
	C−O/C−N (285.30 eV)	48.38%	33.17%	27.95%	28.01%
	C=O (287.80 eV)	6.86%	7.15%	7.10%	9.75%
	O−C=O (289.85 eV)	3.89%	3.00%	3.65%	3.19%
O 1s	O−C (530.57 eV)	18.27%	2.81%	17.94%	26.66%
	O=C (532.01 eV)	66.23%	84.47%	74.36%	45.12%
	O−C=O (533.57 eV)	15.51%	12.71%	17.94%	26.66%
N 1s	Pyridinic−N	27.06%	17.35%	10.92%	12.19%
	Quaternary−N	62.81%	73.17%	76.50%	67.82%
	N−Oxides	10.13%	9.48%	12.58%	19.99%

The C content in the different samples increases with the temperature from NGF900–NGF1100, while it remains approximately constant at 1200 °C. At the same time, the sp^2 component in the C 1s peaks of the samples also increases with the temperature. In contrast, the N content in the samples reduces with pyrolysis temperature. Pyridinic-N component decreases with temperature, whilst the N-oxides increases. The oxygen content fluctuates within a particular range in all samples.

The composition trend was reasonably smooth from NGF900–1100, but “broken” at NGF1200. Almost two times rise is observed for N-oxide peak, and essential drop for O=C,

from average 75% to 45% on the temperature increase from 1100 to 1200 °C. Also an increase was noticed for C−O=C (O 1s) peak.

Overall, the pyrolysis temperature promoted a decrease in the N content of the samples, increasing the graphitization degree, as reported before.[2] However, the O content fluctuated from lower to higher proportions with increased pyrolysis temperatures.

The as-prepared multi-layer graphene-based films roughness and thickness were investigated by Atomic Force Microscopy (AFM). Figure S4 shows AFM images of NGF900, NGF1000, NGF1100 and NGF1200 films which were scratched to determine their thicknesses. Independent cross-section measurements ($n = 6$) on the acquired images of the NGF900, NGF1000, NGF1100 and NGF1200 samples reveal an average particle size of 29.3 ± 5.7, 20.5 ± 1.7, 30.3 ± 2.4 and 45.3 ± 3.5 nm, respectively. The measured roughness mean square (R_q) of all samples is approximately 1.5 nm, indicating very flat and homogeneous surface films.

S3 Steady State Spectroscopy

S3.1 Steady State Spectra Modeling

For a multi-layer graphene-based film or any other semitransparent film, the transmittance in a single passage of light would be $\exp(-\alpha d)$, where α is the absorption coefficient of the material and d is the film thickness. However, part of the light will be reflected from the film surface as the light enters the sample, and part of the light is reflected at the other side of the film, which is the substrate interface in most cases. Furthermore, the reflection will result in the light interference, which affects the measured intensities of the transmitted and reflected light. The reflectance from the surface or at the interface depends on refractive index, which need to be taken into account to model the transmittance and reflectance spectra. Typically, the modeling is done using transfer matrix method (TMM).[3,4] The method allows to calculate the transmittance and reflectance spectra for know film thickness d, and the

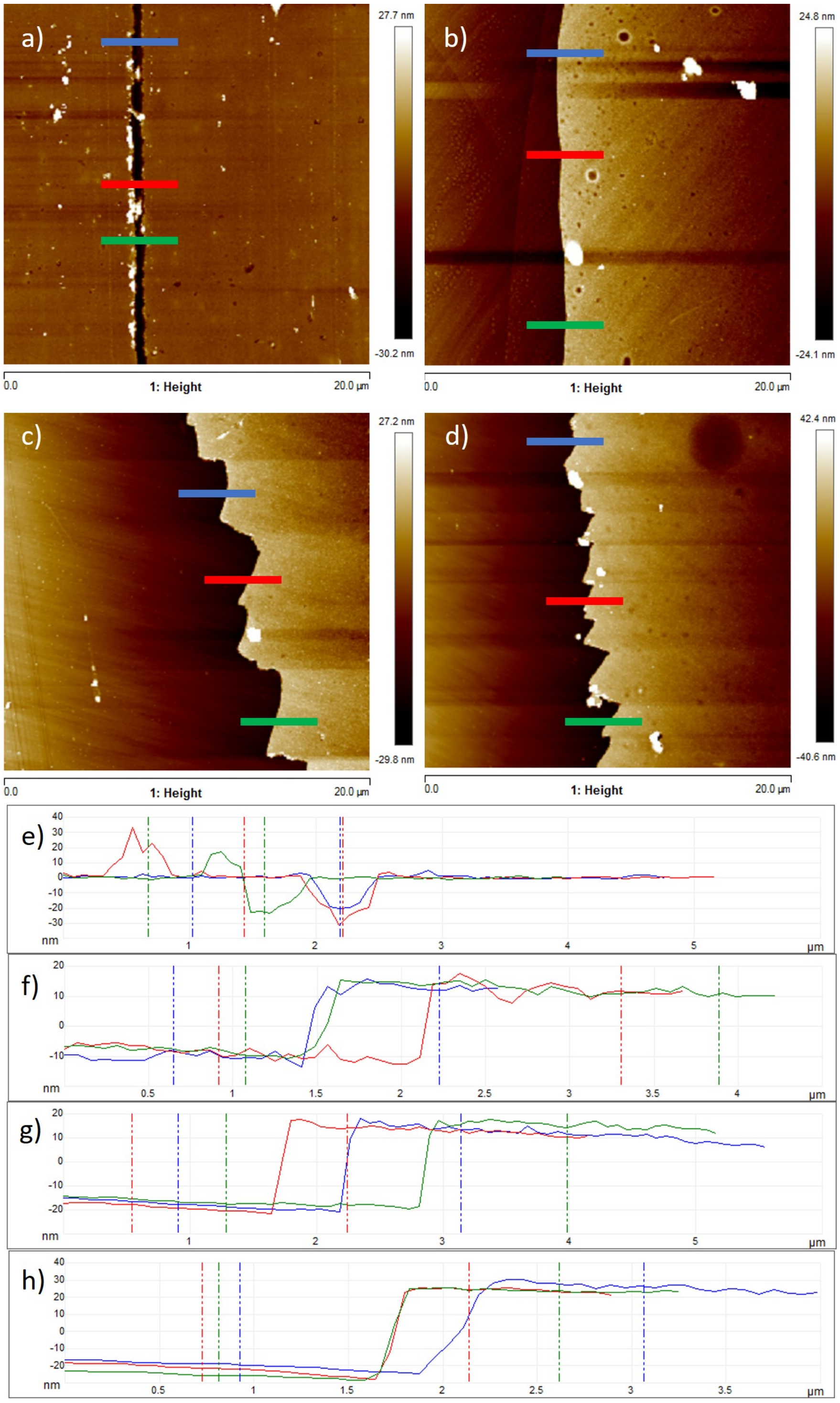

Figure S4: AFM images of the NGF900 (a), NGF1000 (b), NGF1100 (c) and NGF1200 (d) films. Cross-section measurements of the different samples (e-f) are marked on blue, red and green lines with coincident colors.

complex refractive index of the film, $\tilde{n} = n + ik$, where n is the refractive index of the material and k is the extinction coefficient related to the absorption coefficient as $\alpha = \frac{2\pi}{\lambda}k$ where λ is the wavelength. In our case the film is deposited on a quartz (fused silica) substrate for which the refractive index n_s also must be known. We used Sellmeier dispersion equation with coefficients originally reported by Melitson,[5] but slightly adjusted to reproduce better the measured transmittance and reflectance spectra of our substrates. The transmittance, T, and reflectance, R, coefficient of a thin film with (complex) refractive index $\tilde{n}$ deposited on a transparent substrate were calculated using Python library kindly provided by Steve Byrnes and called `tmm`.[4] We assumed a homogeneous photo-induced change of both n and k when modeling photo-responses of the samples. This assumption is reasonable as our film thickness (typically 20–60 nm, see Table S2 below) are much smaller than the wavelength.

Although the complex refractive index is used to calculate optical properties of media, the physical properties are better presented by complex dielectric function $\varepsilon = \varepsilon_1 + i\varepsilon_2$, where $\varepsilon_1 = \Re(\varepsilon)$ and $\varepsilon_2 = \Im(\varepsilon)$. In particular, dielectric properties of graphene can be approximated reasonably well by the so-called Drude-Lorentz (D-L) model[6–8]

$$\varepsilon(E) = \varepsilon_\infty - \frac{E_D^2}{E^2 + iE\Gamma_D} + \frac{A_L^2}{E_L^2 - E^2 - iE\Gamma_L} \tag{S3}$$

where $E = h\nu$ is the photon energy (for measurements in the wavelength domain, $h\nu = hc/\lambda$), ε_∞ is the high frequency dielectric constant, A, E_L and Γ_L are the strength, resonance energy and the width (damping) of the Lorentz band due to $\pi \to \pi^*$ transition, and E_D and Γ_D are the energy and damping of the Drude component, which describes dielectric properties of the free electrons or electron plasma. This presents the optical properties of multi-layer graphene-based films as superposition of three factors, (i) the Drude component due to free carriers or plasmon-type effect, (ii) the Lorentz component due to electronic transitions ($\pi \to \pi^*$ transition) with transition energy corresponding to the UV range, and (iii) a contribution of high frequency transitions (above the Lorentz band) which are presented by

ε_∞.

The relation between the complex refractive index and dielectric function is $\tilde{n} = \sqrt{\varepsilon}$. Therefore, $\tilde{n}$ is known if ε is known, and the measurable transmittance (T) and reflectance (R) spectra of the multi-layer graphene-based film samples can be calculated for given (complex) dielectric function using TMM approach. In other words, the model T and R spectra can be calculated for given film thickness, d, and six D-L parameters, ε_∞, A_L, E_L, Γ_L, E_D and Γ_D.

It can be noted that for each pair of T and R spectra, seven parameters are required to fit the measured spectra. However, at least some of the parameters can be expected to have the same values for different samples, and for a series of reasonably similar graphene compositions and low doping concentrations, the D-L parameters can be expected to be the same which leaves the thickness d to be the only parameter varying from the sample to sample. We measure T and R for each sample in the series and fit the data globally to obtain a set of D-L parameters common for all samples and thicknesses for each sample. There were two samples for each of the four pyrolysis temperatures. The difference between the samples pyrolyzed at the same temperature was minor.

The measured T and R spectra were fitted to estimate the sample thicknesses and D-L parameters. The in-house developed fit program (in Python `fit_TR.py`, see Section S5) uses `tmm`, `numpy`, `scipy` and `lmfit` libraries for calculations.[4,9,10] The program works by calculating the few loaded spectra into dielectric function spectra $\varepsilon(\lambda)$ for each sample, and subsequently converting them to complex refractive index spectra $\tilde{n}(\lambda)$. Then the TMM method is utilized to calculate model T and R spectra for each sample, and calculates the standard deviation of the measured data from the model, which is used as internal parameter for the data fitting (in `lmfit` library).

Since the quality of the T and R spectra was different, a weight factor was introduced when summing up residuals from different type of spectra. The reported results were obtained with weight factors 1 and 0.1 applied to T and R spectra, which accounts for the fact that the T spectra were roughly 10 time more accurate. This discrepancy was attributed to

the low accuracy of the reflectance measurements, at shorter wavelengths due to the reference mirror, as explained in Section S1. A so-called global fitting was utilized which means the spectra of the whole series of samples, NGF900, NGF1000, NGF1100 and NGF1200, were loaded and fitted using different combination of global (common to all samples) and local (individual to each sample) parameters. The fit with common set of D-L parameters delivered the most stable and consistent results with negligible loss of fit accuracy for individual spectra, and this result is reported in the steady state spectra section of the main text.

The model spectra of the real and imaginary parts of the dielectric function, with their D-L components are shown in Figure S5. The dielectric function spectra of the samples in this paper compared to those reported for pyrolytic graphene deposited by chemical vapor deposition (CVD) method[6] are presented in Figure S6. The difference in the amplitudes may be attributed to a somewhat different density of the films, or an average distance between graphene sheets. Aslo, the CVD films has somewhat narrower Lorentz band (at 288 nm). Presumably, pyrolysis results in larger variation of graphene sheets sizes and arrangements, which leads to some variation in the Lorentz band positions.

For the multi-layer graphene-based film reference used in this work (solid lines), the peak of the imaginary part of the dielectric function is at approx. 4.3 eV at the lower limit (≈288 nm) which is within the range of previously reported values, 4–4.6 eV,[11–13] and also in agreement with the CVD graphene spectra shown in Figure S6. There is a report suggesting that the peak of real part of the dielectric function should be at approx. 3 eV (≈413 nm)[11] which is in agreement with the spectra shown in Figure S6.

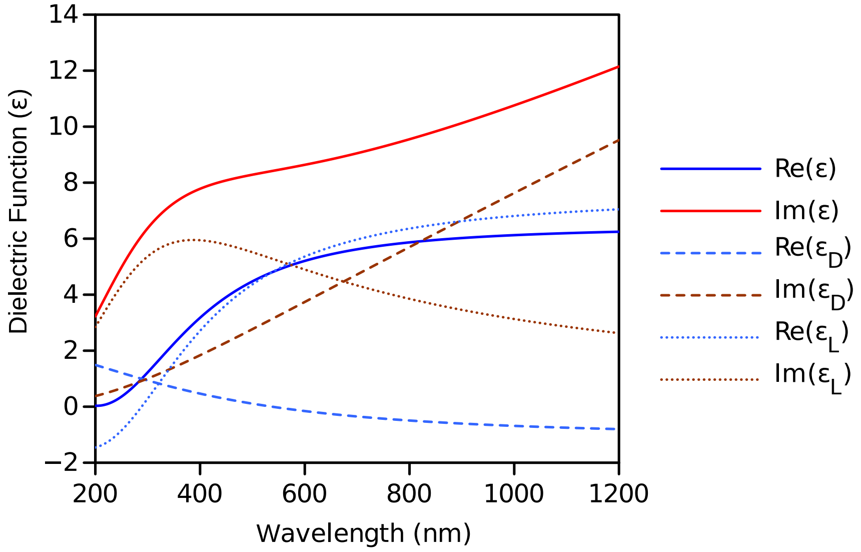

Figure S5: Dielectric function of the NGF900-1200 samples obtained from the global fit of the T and R spectra. The solid line represents the total real (blue) and imaginary (red) parts, while the dashed and dotted lines represent the Drude, ε_D, (including ε_∞) and Lorentz, ε_L, components, respectively.

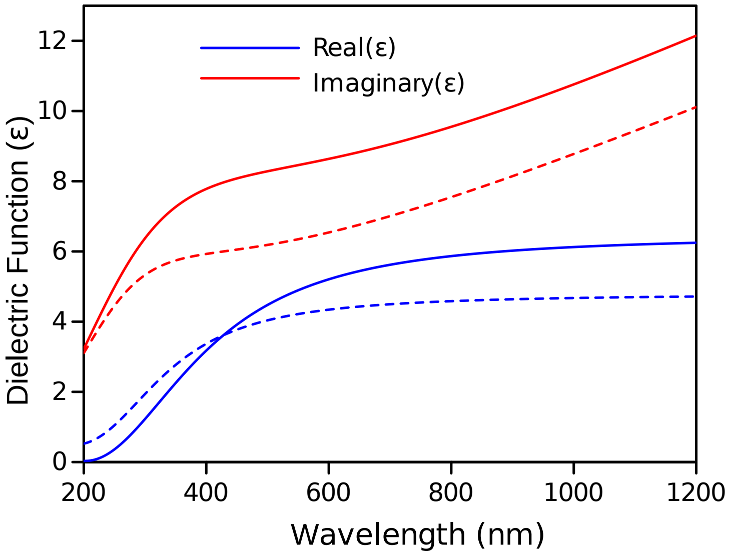

Figure S6: Comparison of the real (blue) and imaginary (red) part of the dielectric function obtained for the sample series in this work (solid lines) and reported for 20 layer graphene prepared by chemical vapor deposition method (dashed lines).[6]

S3.2 Film Thickness Modeling

The film thicknesses estimated, by AFM and optical measurements (T and R) in Table S2 are similar with minor differences due to the acquisition method; AFM revealed the "local" thickness of a scratch, while optical measurements deliver an average thickness from an area of a few squared millimeters. For example, the AFM thickness of NGF1000 was approx. 21 nm, while the optically estimated thickness was approx. 31 nm. However, the trend in the sample thickness variation with the temperature is the same for both estimation methods. The optically estimated thicknesses will be used for transient absorption data fittings since it relies on the same optical properties of the samples as used in the following analysis.

Table S2: Film thicknesses (in nm) obtained from the spectra global fit with D-L fit parameters listed in Table 2.

Temperature, °C	Series 1	Series 2
900	19.1 ± 0.1	19.1 ± 0.1
1000	30.0 ± 0.2	32.5 ± 0.2
1100	44.2 ± 0.2	42.4 ± 0.2
1200	64.0 ± 0.3	65.4 ± 0.3

S4 Transient Absorption Spectroscopy

S4.1 Analysis of Transient Measurements

Standard pump-probe measurements were carried out by detecting both transmitted and reflected light, which will be referred to as transient transmittance (TT) and transient reflectance (TR), respectively. In both cases, the relative change of the probe light intensity was measured and recalculated to "transient absorbance" signals, $\triangle A_T$ and $\triangle A_R$, respec-

tively. The transmittance and reflectance changes obtained from the measured data are:

$$\begin{aligned} \triangle T_m(\lambda, t) &= T(\lambda)\left(10^{-\triangle A_T(\lambda,t)} - 1\right) \\ \triangle R_m(\lambda, t) &= R(\lambda)\left(10^{-\triangle A_R(\lambda,t)} - 1\right) \end{aligned} \tag{S4}$$

However, the obtained $\triangle T_m$ and $\triangle R_m$ are not so informative because any changes in the real or imaginary part of $\tilde{n}$ (or ε, respectively), resulted in changes for both $\triangle T_m$ and $\triangle R_m$. Thus, it was more informative to instead monitor the changes in the real and imaginary part of the dielectric function. Assuming a small photo-induced change of dielectric function, one could rely on the linear approximation:

$$\begin{aligned} \triangle T &= T'_{\varepsilon_1}\triangle\varepsilon_1 + T'_{\varepsilon_2}\triangle\varepsilon_2 \\ \triangle R &= R'_{\varepsilon_1}\triangle\varepsilon_1 + R'_{\varepsilon_2}\triangle\varepsilon_2 \end{aligned} \tag{S5}$$

where T'_{ε_1}, T'_{ε_2}, R'_{ε_1} and R'_{ε_2} are the partial derivatives of T and R over ε_1 and ε_2, respectively. The derivatives can be evaluated numerically for known Drude-Lorentz model parameters and film thickness (d) which are obtained from the steady state transmittance and reflectance spectra analysis. Eq. (S5) is a system of two linear equations and its solution is:

$$\begin{aligned} \triangle\varepsilon_1 &= \frac{\triangle T_m R'_{\varepsilon_2} - \triangle R_m T'_{\varepsilon_2}}{T'_{\varepsilon_1}R'_{\varepsilon_2} - T'_{\varepsilon_2}R'_{\varepsilon_1}} \\ \triangle\varepsilon_2 &= \frac{\triangle R_m T'_{\varepsilon_1} - \triangle T_m R'_{\varepsilon_1}}{T'_{\varepsilon_1}R'_{\varepsilon_2} - T'_{\varepsilon_2}R'_{\varepsilon_1}} \end{aligned} \tag{S6}$$

At this step, the experimental data, $\triangle A_T(\lambda, t)$ and $\triangle A_R(\lambda, t)$, could be recalculated to $\triangle\varepsilon_1(\lambda, t)$ and $\triangle\varepsilon_2(\lambda, t)$ for known $T(\varepsilon)$ and $R(\varepsilon)$. There would be no fitting involved and from this point of view we could derive three equivalent presentations of the experimental results: $(\triangle A_T, \triangle A_R) \Leftrightarrow (\triangle T, \triangle R) \Leftrightarrow (\triangle\varepsilon_1, \triangle\varepsilon_2)$. However, in practice, the spectra $\triangle A_T(\lambda)$ and $\triangle A_R(\lambda)$ at characteristic delay times, or their decay associated spectra (DAS) from primary fittings, were recalculated to the spectra of $\triangle\varepsilon_1(\lambda)$ and $\triangle\varepsilon_2(\lambda)$ (see the main text for details).

Finally, to better understand the phenomena behind the photo-induced change of the dielectric function, the spectra of $\triangle\varepsilon_1(\lambda)$ and $\triangle\varepsilon_2(\lambda)$ were fitted to the changes in D-L parameters: $\triangle\varepsilon_\infty$, $\triangle A_L$, $\triangle E_L$, $\triangle\Gamma_L$, $\triangle E_D$ and $\triangle\Gamma_D$ using eq. (S3), namely

$$\triangle\varepsilon(\lambda) = \varepsilon(\lambda, p + \triangle p) - \varepsilon(\lambda, p) \tag{S7}$$

where p is the set (vector) of D-L parameters, $p = (\varepsilon_\infty, A_L, E_L, \Gamma_L, E_D, \Gamma_D)$, $\triangle p = (\triangle\varepsilon_\infty, \triangle A_L, \triangle E_L, \triangle\Gamma_L, \triangle E_D, \triangle\Gamma_D)$, respectively, and $\varepsilon()$ is calculated according to eq. (S3). This was the final step of the TT and TR data treatment.

In conclusion, the key steps for the collection and analysis of the spectroscopy data and schematically outlined in Figure 4, are:

1. measure steady state transmittance, T, and reflectance, R, spectra of a series of samples;

2. fit the spectra to the D-L model, eq. (S3) and using TMM approach to account for the sample thickness; this results in a set of D-L parameters (common to all the samples) and thickness evaluations (for each sample), ε_∞, A, E_L, Γ_L, E_D, Γ_D and d, respectively;

3. conduct transient absorption measurements in transmittance and reflectance modes, which deliver 2D arrays of spectroscopy data, $\triangle A_T(\lambda, t)$ and $\triangle A_R(\lambda, t)$, respectively;

4. fit the data to determine characteristic time constants and characteristic spectra corresponding to specific intermediate state; this results in pairs of spectra, $\triangle A_T(\lambda)$ and $\triangle A_R(\lambda)$, to be analysed further;

5. recalculate the $\triangle A_T(\lambda)$ and $\triangle A_R(\lambda)$ spectra to $\triangle\varepsilon(\lambda)$ spectra using previously determined D-L parameters and thicknesses (step 2) to evaluate T'_{ε_1}, T'_{ε_2}, R'_{ε_1} and R'_{ε_2} and solve eq. (S6);

6. fit the $\triangle\varepsilon(\lambda)$ spectra to obtain corresponding changes of D-L parameters ($\triangle\varepsilon_\infty$, $\triangle A_L$, $\triangle E_L$, $\triangle\Gamma_L$, $\triangle E_D$ and $\triangle\Gamma_D$), eq. (S7).

The data were processed using in-house developed programs written in Python and using `numpy`[9] and `tmm` libraries (see Section S5).

S4.2 Transient Absorption Measurements

The TT and TR decays at 650 nm in logarithmic scale of the samples pyrolyzed at 900-1200 °C are presented in Figures S7–S10. The TT and TR decays for the 1000 °C sample in sub-logarithmic scale compared to the undoped GTF sample are presented in the main text (Figure 2).

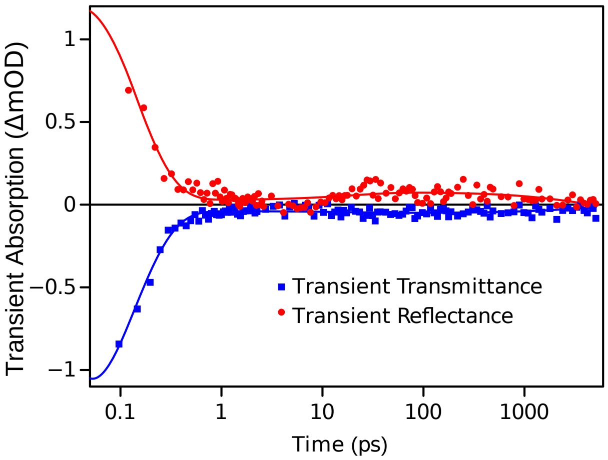

Figure S7: TT and TR decay profiles of the sample pyrolyzed at 900 °C monitored at 650 nm. The time scale is logarithmic.

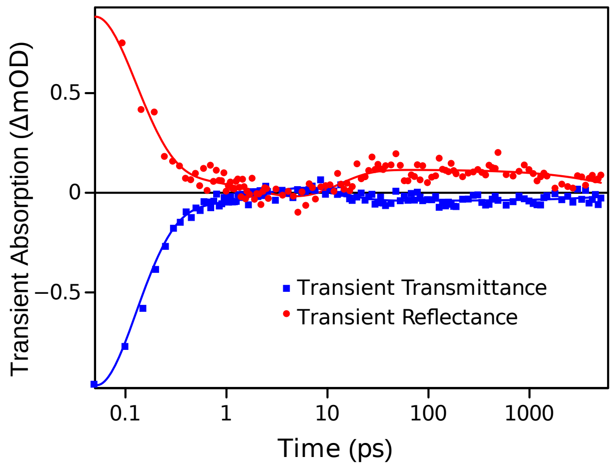

Figure S8: TT and TR decay profiles of the sample pyrolyzed at 1000 °C monitored at 650 nm. The time scale is logarithmic.

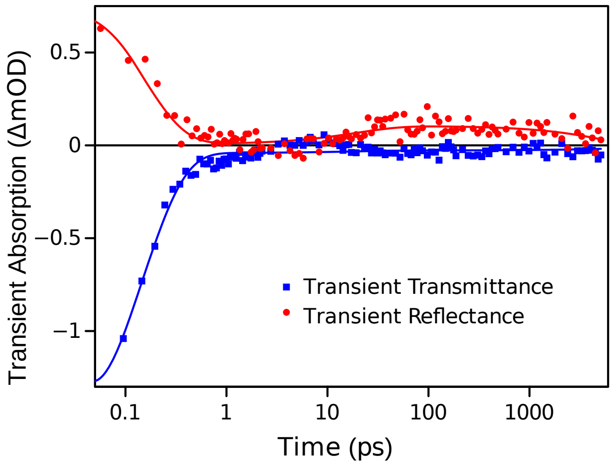

Figure S9: TT and TR decay profiles of the sample pyrolyzed at 1100 °C monitored at 650 nm. The time scale is logarithmic.

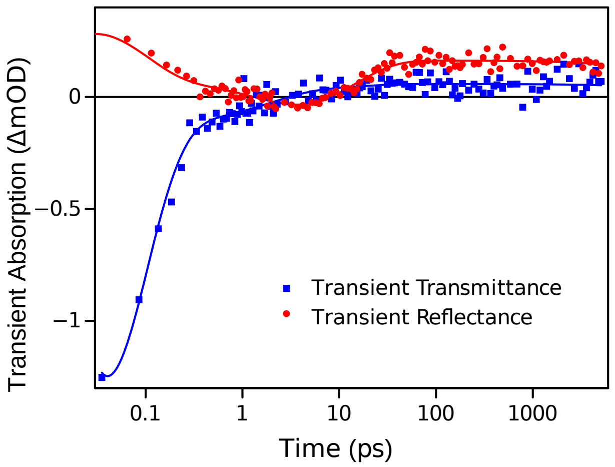

Figure S10: TT and TR decay profiles of the sample pyrolyzed at 1200 °C monitored at 650 nm. The time scale is logarithmic.

Decay associated spectra (DAS) resulting from the global fit of TT and TR measurements of samples pyrolyzed at 900, 1100 and 1200 °C (i.e. NGF900, NGF1100 and NGF1200) are presented in Figures S11–S13. A similar figure for NGF1000 is presented in the main text (Figure 3).

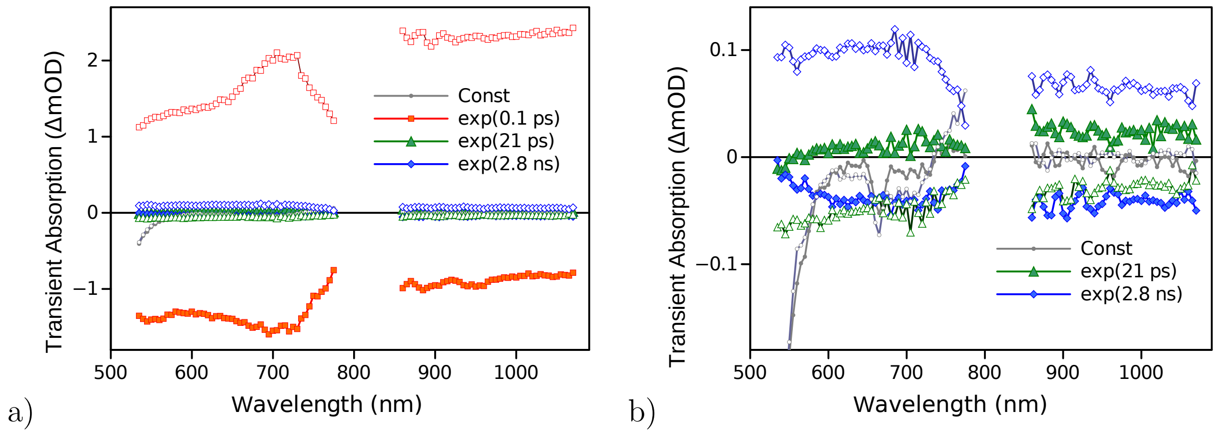

Figure S11: DAS resulting from the global fit of the TT and TR spectra for the NGF900 sample. The transmittance and reflectance DAS are indicated by filled symbols and open symbols respectively. Plots (a) and (b) represent the same data but in plot (b) the scale is magnified to highlight the second wave.

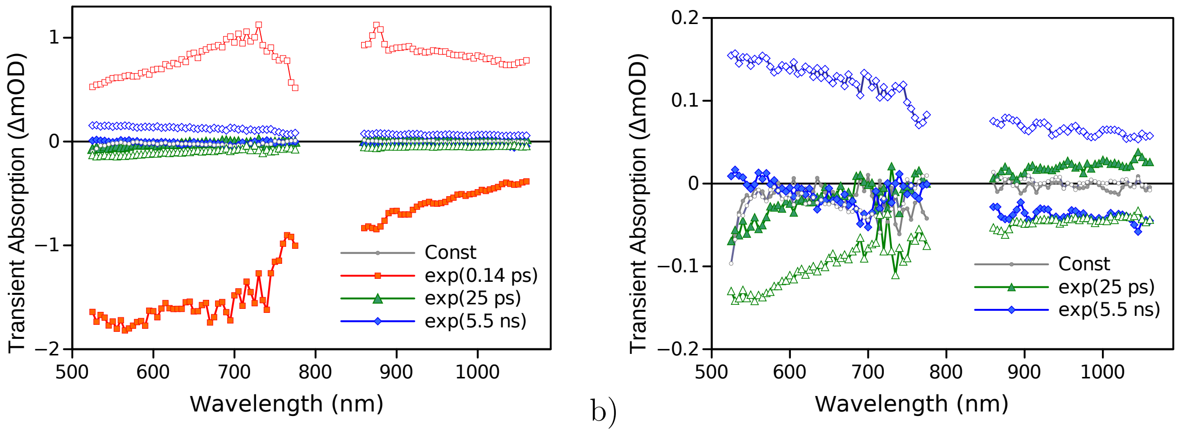

a) b)

Figure S12: DAS resulting from the global fit of the TT and TR spectra for the NGF1100 sample. The transmittance and reflectance DAS are indicated by filled symbols and open symbols respectively. Plots (a) and (b) represent the same data but in plot (b) the scale is magnified to highlight the second wave.

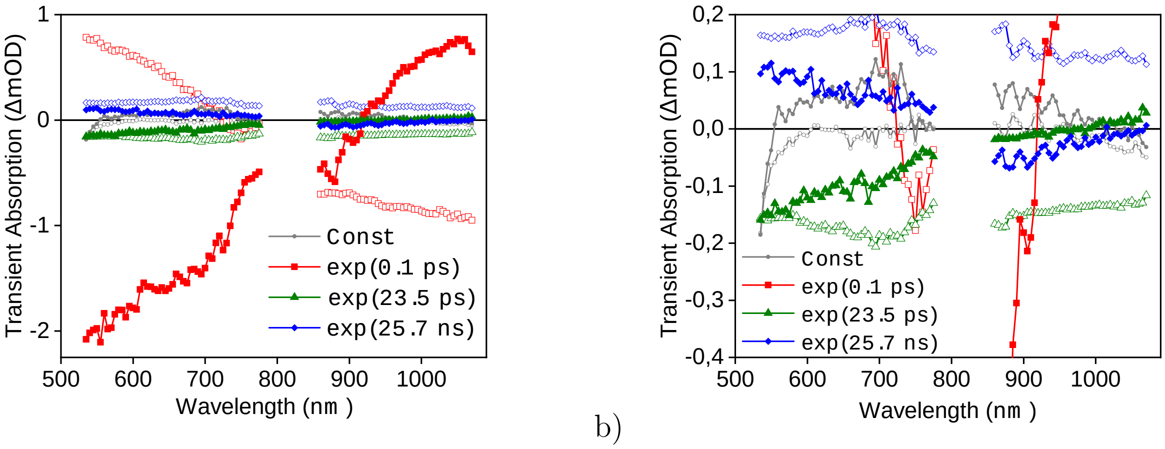

a) b)

Figure S13: DAS resulting from the global fit of the TT and TR spectra for the NGF1200 sample. The transmittance and reflectance DAS are indicated by filled symbols and open symbols respectively. Plots (a) and (b) represent the same data but in plot (b) the scale is magnified to highlight the second wave.

S4.3 Excitation Density, Excitation Wavelength and Monitoring Wavelength Dependence

The excitation density, excitation wavelength and monitoring wavelength dependencies of the undoped GTF and doped NGF1100 films were explored. The excitation density dependence measurements were made at 500 nm excitation to monitor the decays of the multi-layer graphene-based films at 700 nm in the reflectance mode. The reflectance mode was used to ensure clear measurement of the "second wave". From these decays, the intensities of the "first wave" (at approx. 0.1 ps) and the "second wave" (at approx. 40 ps) are plotted at three different excitation density dependencies of 0.05, 0.1 and 0.2 mJ/cm^2. The resulting excitation density dependencies are shown in Figure S14.

The NGF1100 film has both the "first wave" and "second wave" with linear dependencies as a function of power. However, it should be noted that the GTF film has only the "first wave" with a linear dependence while the "second wave" was absent. The "second wave" represented for GTF was measured at the same time scale as the "second wave" of NGF1100, to highlight the presence of the "second wave" in the NGF series.

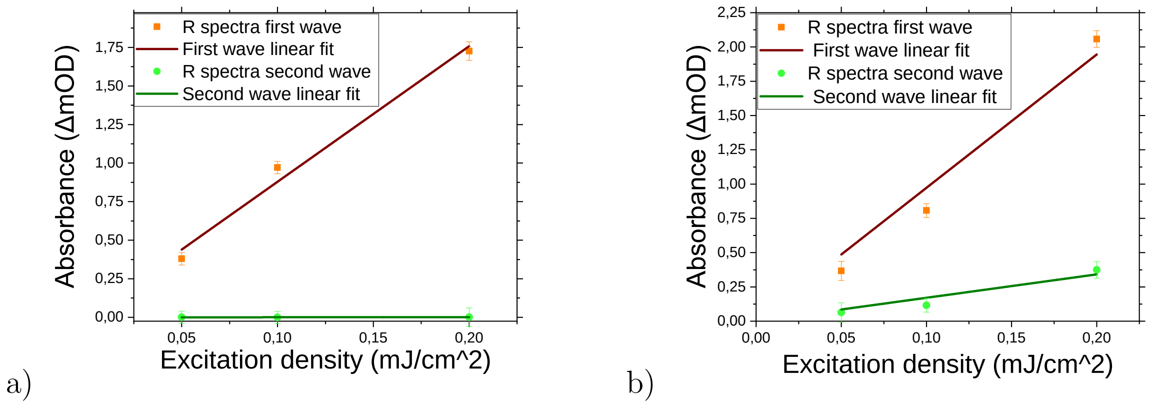

Figure S14: The excitation density dependencies of (a) GTF (b) NGF1100 at 500 nm excitation, monitored at 700 nm.

The excitation wavelength dependence measurements were made at 0.1 mJ/cm^2 excitation density to monitor the decays of the multi-layer graphene-based films at 700 nm. From

these decays, the intensities of the “first wave” (at approx. 0.1 ps) and the “second wave” (at approx. 40 ps) were plotted at three different wavelengths (energies) of 320 (3.875), 500 (2.48) and 640 (1.94) nm (eV). The resulting excitation wavelength dependencies are shown in Figure S15.

The excitation wavelength dependencies did not show any significant changes to the photophysics of the undoped GTF and the N-doped NGF1100 at different excitation wavelengths (energies). GTF without a “second wave” within noise levels while the NGF1000 has a prominent “second wave”. The differences in the absorbance intensities were mainly due to the differences in amount of photons absorbed by carriers at different excitation wavelengths (energies). Therefore no essential excitation wavelength (energy) dependence was observed for all samples.

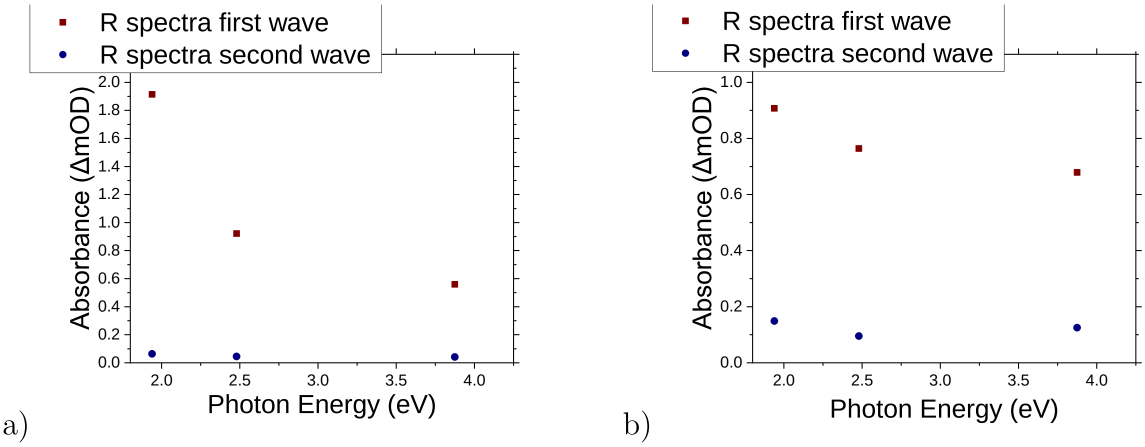

a) b)

Figure S15: The excitation wavelength (energy) dependencies of (a) GTF (b) NGF1100 at 0.1 mJ/cm^2 excitation density, monitored at 700 nm.

The monitoring wavelength dependence measurements were made at 500 nm excitation wavelength and 0.1 mJ/cm^2 excitation density to monitor the decays of the multi-layer graphene-based films. These decays were made at monitoring wavelengths of 600, 750, 900 and 1050 nm to determine how this dependence could affect the lifetimes of the GTF and NGF1100 films. The resulting monitoring wavelength dependencies are shown in Figure S15.

The monitoring wavelength dependencies did not show any significant changes to the

lifetimes of the undoped GTF and the N-doped NGF1100 at 500 nm excitation, which supports our case for the utilization of a global fitting in our model.

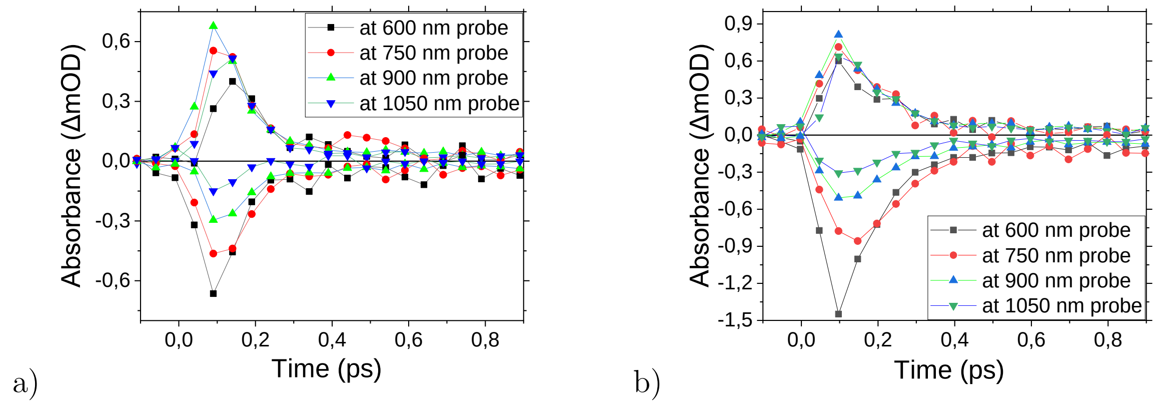

Figure S16: The monitoring wavelength (energy) dependencies of (a) GTF (b) NGF1100 at 500 nm excitation wavelength, and 0.1 mJ/cm^2 excitation density.

S4.4 Comparison of the Transient Absorption Response

All doped samples were measured in reasonably same conditions (same excitation densities) which allows "direct" comparison of the responses spectra and spectra amplitudes. The spectra of the "first wave" (fitted results) are presented in Figure S17. The responses of samples pyrolyzed at 900, 1000, and 1100 °C were rather similar to each other. However, the "first wave" response spectrum of the sample pyrolyzed at 1200 °C differs significantly from that of the samples pyrolyzed at lower temperatures. Also the "second wave" spectrum of the sample pyrolyzed at 1200 °C differs significantly from that of the samples pyrolyzed at lower temperature as will be discussed later.

Next, the $\triangle A_T(\lambda)$ and $\triangle A_R(\lambda)$ spectra were recalculated to $\triangle\varepsilon(\lambda)$ (complex) spectrum, as described above, eq. (S6) and step 5. The calculated changes in dielectric function corresponding to the "first wave" are presented in Figure 5 of the main text.

The "second wave" responses ($\triangle A_T$ and $\triangle A_R$) are presented in Figure S18. The responses are more complex but also show a trend. The calculated changes in the dielectric

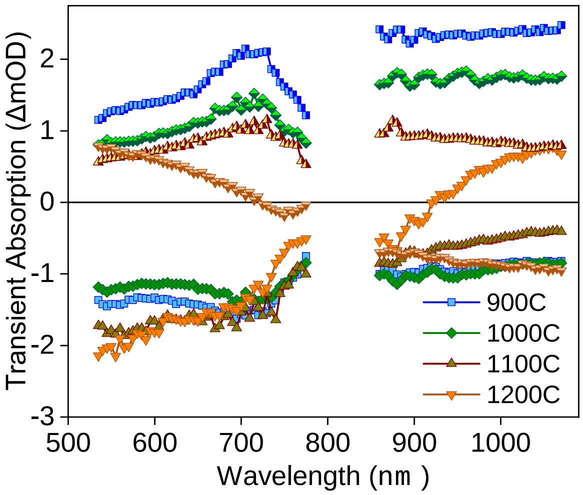

Figure S17: The measured $\triangle A_T$ (whole symbol) and $\triangle A_R$ (split symbol) spectra of the "first wave" of the NGF900–NGF1200 series of samples. The pyrolysis temperatures are 900 (blue square), 1000 (green diamond), 1100 (brown triangle) and 1200 °C (orange triangle).

functions are presented in Figure S19 (refer to the main text Figures 5c and 5d for magnified ε scale). The most drastic change is a strong increase in $\triangle\varepsilon$ at $\lambda < 600$ nm at highest pyrolysis temperature (1200 °C). This is apparently due to strong change in the samples absorbance at $\lambda < 500$ nm, though we do not see the maximum in the measured range. Most probably there is a bleach of the band at 270 nm.

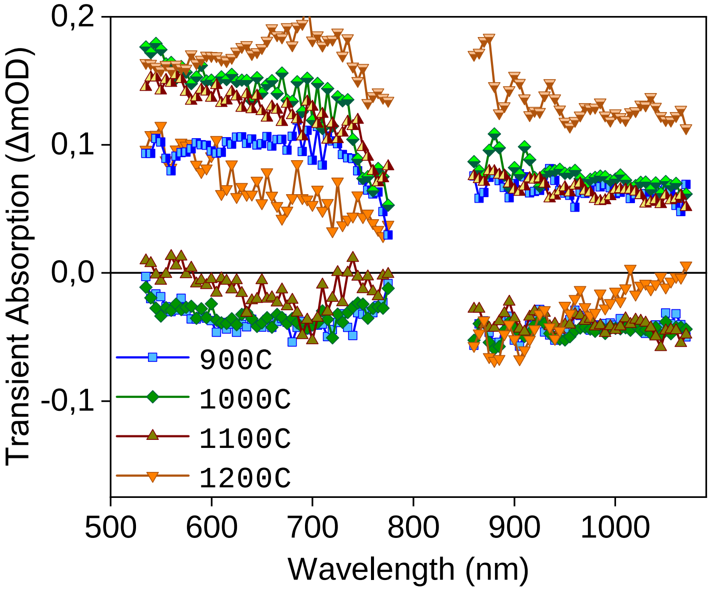

Figure S18: The TA spectra associated with the "second wave" and obtained in transmittance (whole symbol) and reflectance (split symbol) modes, $\triangle A_T$ and $\triangle A_T$, respectively. The pyrolysis temperatures are 900 (blue square), 1000 (green diamond), 1100 (brown triangle) and 1200 °C (orange triangle).

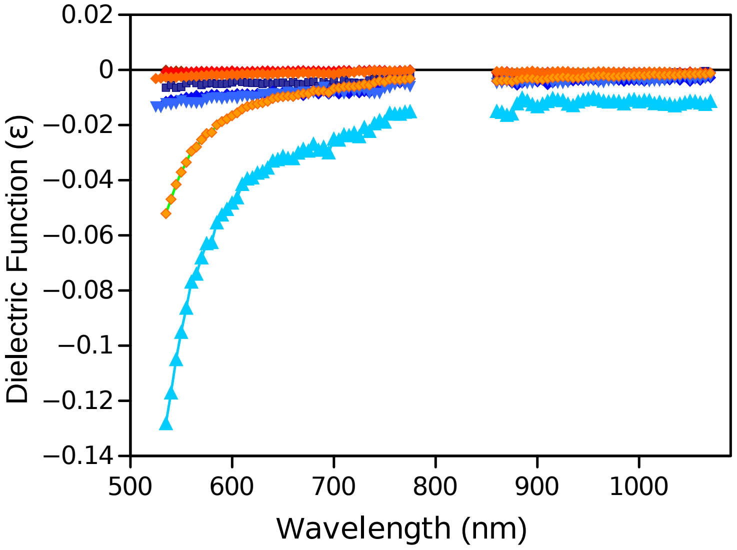

Figure S19: The spectra of real (blue) and imaginary part (red) of the dielectric function corresponding to the "second wave". The pyrolysis temperatures are 900, 1000, 1100 and 1200 °C and the darker color corresponds to lower temperature.

The decay time constant for the "first wave" (in the mono-exponential approximation) is 100–150 fs for all samples. The rise time constant for the "second wave" is 20–30 ps for all the samples (no temperature dependence). The lifetime of the "second wave" increases from 3 to roughly 30 ns as the pyrolysis temperature increases. With the exception of 1200 °C, there is a trend for the "second wave" with the real and imaginary parts of the dielectric function (Figure S19) which is reversed compared to the "first wave", a higher pyrolysis temperature leads to stronger response.

S4.5 The Effect of D-L Parameter Changes on the Transient Absorption

The changes in D-L model parameters have different effects on the $\varepsilon(\lambda)$ spectra and consequently on measured TT ($\triangle A_T(\lambda)$) and TR ($\triangle A_R(\lambda)$) responses. To illustrate the effect of each of D-L parameter change on the measured responses Figure S20 presents the calculated responses $\triangle A_T(\lambda)$ and $\triangle A_R(\lambda)$ assuming 1 % increase of only one of the parameters at a time for the sample with dielectric function set by eq. (1) with parameters reported in Table 2 with thickness 30 nm corresponding to NGF1000 sample.

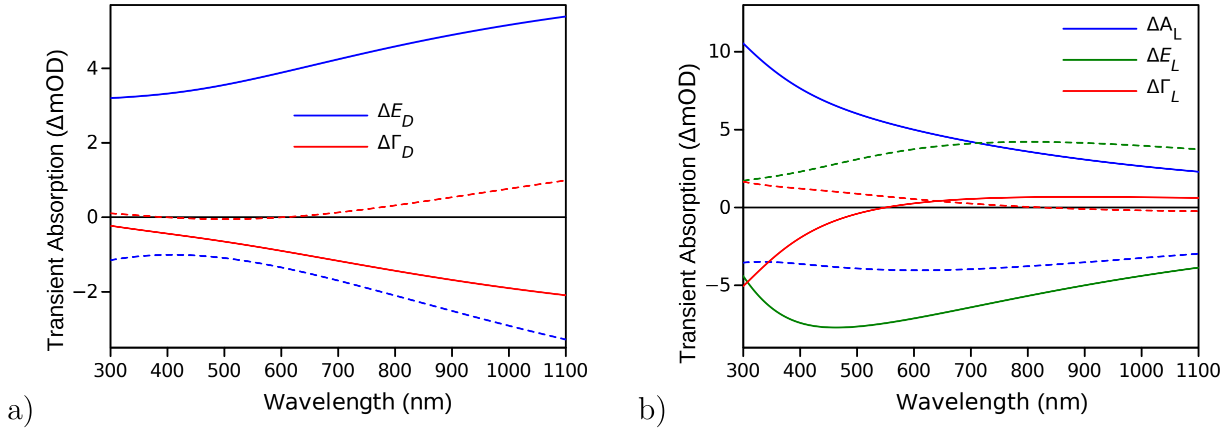

Figure S20: The $\triangle A_T(\lambda)$ (the solid lines) and $\triangle A_R(\lambda)$ (the dashed lines) calculated for sample NGF1000 assuming 1 % increase of (a) Drude energy ($\triangle E_D$) or Drude dumping ($\triangle \Gamma_D$), and (b) Lorentz band intensity ($\triangle A_L$), or resonance energy ($\triangle E_L$), or dumping ($\triangle \Gamma_L$).

S4.6 The Photo-Induced Change of the Dielectric Function

Figures S21–S23 show the dielectric functions change corresponding to the "first" and "second waves" for samples pyrolyzed at 900, 1100 and 1200 °C (i.e. NGF900, NGF1100 and NGF1200) and the fits to D-L parameter photo-induced change. A similar figure for 1000 °C sample is in the main text (Figure 6).

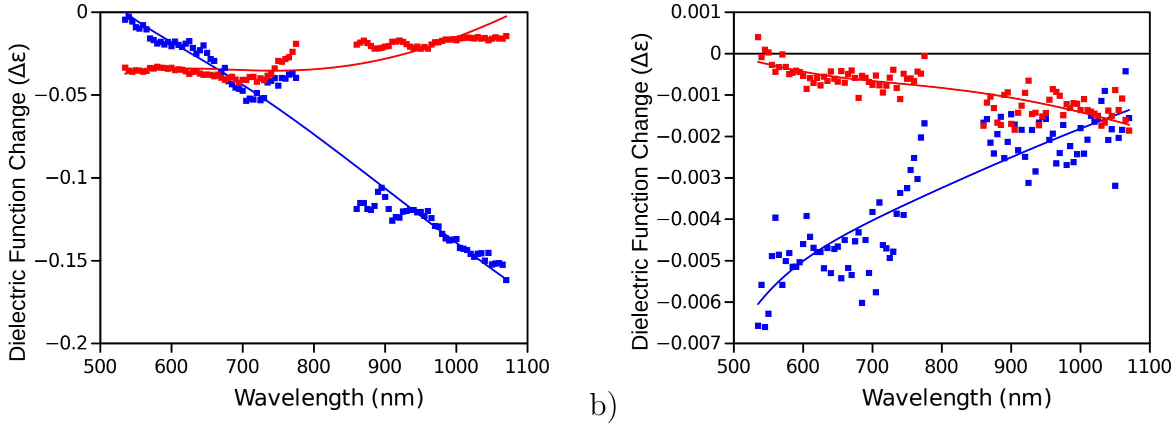

a) b)

Figure S21: The fits of the real (blue) and imaginary (red) part of the dielectric function change for the "first wave" (a) and the "second wave" (b) of the NGF900 sample (The symbols are from the raw measured TT and TR responses and the lines are from the fitting results).

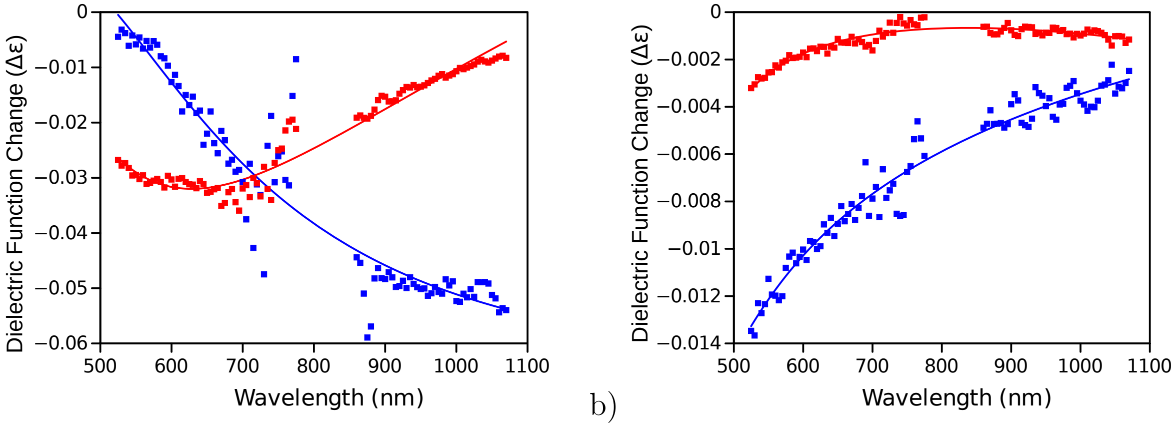

a) b)

Figure S22: The fits of the real (blue) and imaginary (red) part of the dielectric function change for the "first wave" (a) and the "second wave" (b) of the NGF1100 sample (The symbols are from the raw measured TT and TR responses and the lines are from the fitting results).

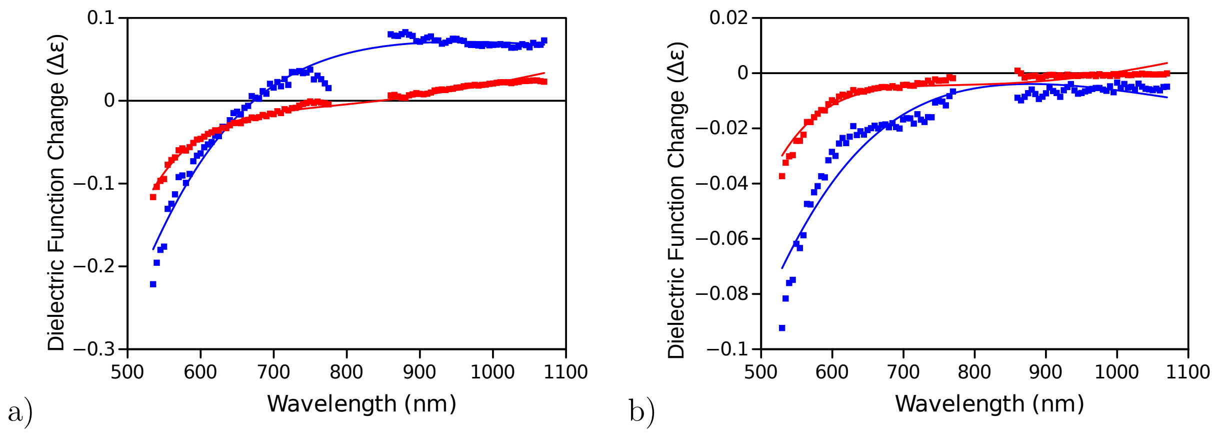

Figure S23: The fits of the real (blue) and imaginary (red) part of the dielectric function change for the “first wave” (a) and the “second wave” (b) of the NGF1200 sample (The symbols are from the raw measured TT and TR responses and the lines are from the fitting results).

S5 Data Analysis Software

Three command line programs were developed to analyse the data.

Steady State Spectra Fit. The program `fit_TR.py` was designed to fit steady state T and R spectra. An example of the use case is

```
> python3 fit_TR.py -a 2 -w 0.1 --gdump GTF_TR_cut230.txt Chl900_TR_cut225.txt
Chl1000_TR_cut226.txt
```

which uses 2 data point averaging (`-a 2`), relative weight factor for the R spectra is 0.1 (`-w 0.1`), calculations are done assuming global Drude dumping (`--gdump`), and it reads data to be fitted from `GTF_TR_cut230.txt Chl900_TR_cut225.txt Chl1000_TR_cut226.txt`. By default it outputs results of the fit (D-L parameters and calculated spectra) to `fit_TR.txt` file. More command line options are available on `fit_TR.py --help`.

Conversion of TA Spectra to Dielectric Function Change. The second program is `L-D_fit_d-eps.py`, and it calculates $\triangle\varepsilon$ out of $\triangle A_T$ and $\triangle A_R$ for given D-L parameters.

The use case example is

```
> python3 L-D_fit_d-eps.py pars_GTF-2.txt -t GTF_dT-dR_50fs.txt
```

This will use D-L model parameters and film thickness saved in file `pars_GTF-2.txt` to calculate the T and R derivatives, and convert the measured $\triangle A_T$ and $\triangle A_R$ to $\triangle\varepsilon_1$ and $\triangle\varepsilon_2$ ($\triangle\varepsilon = \triangle\varepsilon_1 + i\triangle\varepsilon_2$) using algorithm described above. By default, the results are saved in file `d_eps.txt`.

Fitting of the Dielectric Function. The third program, `d-eps_fit.py`, takes complex dielectric function photoinduced change $\triangle\varepsilon$ and D-L parameters, and fit the change of the function by changing the D-L parameters. An example of the program use is

```
> python3 d-eps_fit.py Chi1000-1_d_eps_2nd.txt --fix_D_E
```

which reads D-L parameters and (real and imaginary parts of) dielectric function from file `Chi1000-1_d_eps_2nd.txt` and fits it to the change of the D-L parameters except of Drude energy E_D which has a fixed value (`--fix_D_E`). In the same manner any combination of D-L parameters can be fixed (`d-eps_fit.py --help` prints out help information). The format of the input file (file `Chi1000-1_d_eps_2nd.txt` in the example) is the same as output format of `L-D_fit_d-eps.py` program, therefore these two programs can be used in a chain.

All the programs are open source software and can be obtained on request.

References

(1) Jerng, S.-K.; Seong Yu, D.; Hong Lee, J.; Kim, C.; Yoon, S.; Chun, S.-H. Graphitic Carbon Growth on Crystalline and Amorphous Oxide Substrates using Molecular Beam Epitaxy. *Nanoscale Res. Lett.* **2011**, *6*, 565, DOI: 10.1186/1556-276X-6-565.

(2) He, J.; Anouar, A.; Primo, A.; García, H. Quality Improvement of Few-Layers Defective Graphene from Biomass and Application for H_2 Generation. *Nanomaterials* **2019**, *9*, 895, DOI: 10.3390/nano9060895.

(3) Katsidis, C. C.; Siapkas, D. I. General Transfer-Matrix Method for Optical Multilayer Systems with Coherent, Partially Coherent, and Incoherent Interference. *Appl. Opt.* **2002**, *41*, 3978–3987, DOI: 10.1364/AO.41.003978.

(4) Byrnes, S. J. Multilayer Optical Calculations. 2016; `https://arxiv.org/abs/1603.02720`, (accessed 2023-01-16).

(5) Malitson, I. H. Interspecimen Comparison of the Refractive Index of Fused Silica. *J. Opt. Soc. Am. 55*, 1205–1209, DOI: 10.1364/JOSA.55.001205.

(6) Dovbeshko, G. I.; Romanyuk, V. R.; Pidgirnyi, D. V.; Cherepanov, V. V.; Andreev, E. O.; Levin, V. M.; Kuzhir, P. P.; Kaplas, T.; Svirko, Y. P. Optical Properties of Pyrolytic Carbon Films Versus Graphite and Graphene. *Nanoscale Res. Lett.* **2015**, *10*, 234, DOI: 10.1186/s11671-015-0946-8.

(7) Song, B.; Gu, H.; Zhu, S.; Jiang, H.; Chen, X.; Zhang, C.; Liu, S. Broadband Optical Properties of Graphene and HOPG investigated by Spectroscopic Mueller Matrix Ellipsometry. *Appl. Surf. Sci.* **2018**, *439*, 1079–1087, DOI: 10.1016/j.apsusc.2018.01.051.

(8) Shen, C. C.; Lin, C. T.; Li, L. J.; Liu, H. L. Charge Dynamics and Electronic Structures of Monolayer Graphene with Molecular Doping. *Appl. Phys. Lett.* **2012**, *101*, 111907, DOI: 10.1063/1.4752131.

(9) Harris, C. R.; Millman, K. J.; van der Walt, S. J.; Gommers, R.; Virtanen, P.; Cournapeau, D.; Wieser, E.; Taylor, J.; Berg, S.; Smith, N. J. et al. Array Programming with NumPy. *Nature* **2020**, *585*, 357–362, DOI: 10.1038/s41586-020-2649-2.

(10) Virtanen, P.; Gommers, R.; Oliphant, T. E.; Haberland, M.; Reddy, T.; Cournapeau, D.; Burovski, E.; Peterson, P.; Weckesser, W.; Bright, J. et al. SciPy 1.0: Fundamental Algorithms for Scientific Computing in Python. *Nature Methods* **2020**, *17*, 261–272, DOI: 10.1038/s41592-019-0686-2.

(11) Zhou, X.; Zhao, C.; Wu, G.; Chen, J.; Li, Y. DFT Study on the Electronic Structure and Optical Properties of N, Al, and N-Al Doped Graphene. *Appl. Surf. Sci.* **2018**, *459*, 354–362, DOI: 10.1016/j.apsusc.2018.08.015.

(12) Olaniyan, O.; Maphasha, R.; Madito, M.; Khaleed, A.; Igumbor, E.; Manyala, N. A Systematic Study of the Stability, Electronic and Optical Properties of Beryllium and Nitrogen Co-Doped Graphene. *Carbon* **2018**, *129*, 207–227, DOI: 10.1016/j.carbon.2017.12.014.

(13) Rani, P.; Dubey, G. S.; Jindal, V. DFT Study of Optical Properties of Pure and Doped Graphene. *Phys. E* **2014**, *62*, 28–35, DOI: 10.48550/arXiv.1404.4759.
